# SARS-CoV-2 infection of human cortical cells is influenced by the interaction between aneuploidy and biological sex: insights from a Down syndrome in vitro model

**DOI:** 10.1007/s00401-025-02895-2

**Published:** 2025-05-30

**Authors:** Maria I. Lioudyno, Evgueni A. Sevrioukov, Gema M. Olivarria, Lauren Hitchcock, Dominic I. Javonillo, Sydney M. Campos, Isabel Rivera, Sierra T. Wright, Elizabeth Head, Juan Fortea, Thomas Wisniewski, A. Claudio Cuello, Sonia Do Carmo, Thomas E. Lane, Jorge Busciglio

**Affiliations:** 1 Department of Neurobiology and Behavior, Institute for Memory Impairments and Neurological Disorders, University of California Irvine, Irvine, CA USA; 2 Department of Medicine, Division of Infectious Diseases, UC Irvine School of Medicine, Irvine, CA USA; 3 Samueli School of Engineering, University of California Irvine, Irvine, CA USA; 4 Department of Pathology and Laboratory Medicine, Institute for Memory Impairments and Neurological Disorders, University of California Irvine, Irvine, CA USA; 5 Sant Pau Memory Unit, Department of Neurology, Hospital de La Santa Creu I Sant Pau, Institut d’Investigació Biomèdica Sant Pau (IIB SANT PAU), Facultad de Medicina - Universitat Autònoma de Barcelona, Barcelona, Spain; 6 Centro de Investigación Biomédica en Red de Enfermedades Neurodegenerativas (CIBERNED), Madrid, Spain; 7 Barcelona Down Medical Center, Fundació Catalana de Síndrome de Down, Barcelona, Spain; 8 Departments of Neurology, Pathology and Psychiatry and Center for Cognitive Neurology, New York University Grossman School of Medicine, New York, NY USA; 9 Department of Pharmacology and Therapeutics, McGill University, Montreal, QC Canada; 10 Department of Molecular Biology and Biochemistry, University of California Irvine, Irvine, CA USA

**Keywords:** Trisomy-21, SARS-CoV-2, Interferon system, Sex differences, Primary cortical cultures, Postmortem brain specimens

## Abstract

Individuals with Down Syndrome (DS) represent one of the most susceptible populations for developing severe COVID-19, and a unique human genetic condition for investigating molecular mechanisms underlying susceptibility of neurologically vulnerable individuals to SARS-CoV-2 infection. Human Chromosome-21 (HSA21) triplication in DS causes global transcriptional deregulation, affecting multiple genes that may directly (e.g., TMPRSS2) or indirectly influence the SARS-CoV-2 entry into central nervous system (CNS) cells. The anti-viral immune response may also be altered in cells with trisomy-21 (T21) due to triplication of genes encoding for several interferon receptor subunits and interferon-stimulated genes (ISGs). Here, we demonstrate that human cells derived from fetal cortical specimens and maintained in primary cultures are susceptible to infection with a molecular clone of vesicular stomatitis virus engineered to express the Spike protein of SARS-CoV-2 (VSV-eGFP-SARS-CoV-2) and to authentic SARS-CoV-2. The level of SARS-CoV-2 infectivity in cultures originated from different cortical specimens varied, seemingly depending on ploidy and chromosomal sex of the cells. We confirmed the presence of ACE2 and TMPRSS2 in cultures and found that XY T21 group had the highest TMPRSS2 mRNA levels, which was associated with increased infectivity in XY—compared to XX T21 cultures. The XX T21 cultures exhibited elevated expression of several ISGs (MX1, STAT1, and STAT2) which was associated with lower infectivity. The comparisons of postmortem aged brain specimens revealed reduced ACE2, TMPRSS2, but elevated STAT2 protein levels in individuals with DS and Alzheimer’s disease (DS-AD) compared to control and Alzheimer’s disease (AD) group. Collectively, these results suggest multifactorial regulation of SARS-CoV-2 infectivity in cortical cells that involves ploidy, chromosomal sex, and the expression of genes implicated in regulation of virus entry and anti-viral response as contributing factors.

**Supplementary Information:**

The online version contains supplementary material available at 10.1007/s00401-025-02895-2.

## Introduction

A broad range of COVID-19 symptoms are related to the SARS-CoV-2 ability to affect a plethora of organs and cells in the body. Although over 30% of patients with COVID-19 reportedly experience neurological symptoms [57, 84], the central nervous system (CNS) invasion routes (e.g., via anterograde axonal transport along peripheral nerves [22, 87], by crossing the cribriform plate and dissemination from olfactory epithelium [118] or through the compromised blood–brain barrier [54, 67, 72, 78]), as well as the mechanisms underlying pathogenesis of neurological sequelae of SARS-CoV-2 are still debated [1, 16, 23, 42, 47, 129] and likely present complex combination of multiple and dynamic processes that differ between cases. Among these mechanisms, both direct infection of neural cells with SARS-CoV-2 [13, 21, 100] and indirect effects on the CNS mediated by a systemic immune reaction [1, 43, 97] are proposed, with the former remaining challenging to demonstrate possibly due to a transient nature of active virus infection as well as great diversity in cellular responses and the degree of damage between infected individuals [24, 93, 113].

Although no uniform consensus exists on whether SARS-CoV-2 directly infects neural cells [23, 30, 42, 47, 81, 88], several brain autopsy studies and investigations using iPSC-derived brain organoids and cultures enriched with resident cells of the CNS suggested the SARS-CoV-2 infection of human neurons and/or other cell types in the CNS. Specifically, these studies reported the presence of SARS-CoV-2 protein and/or RNA in brain parenchyma [13, 36, 45, 52, 86, 87, 89, 100, 106, 115, 119, 124] and visualized virus-associated alterations in neurons and glial cells in the postmortem brains of COVID-19 patients [53, 79]. They have also concluded that viral particles are present and replicate within the iPSC-derived brain organoids [115], described a differential susceptibility of iPSC-derived neurons, astrocytes, and microglia to pseudotyped lentiviruses expressing the S protein of SARS-CoV-2 [71], and demonstrated the expression of entry proteins ACE2 and TMPRSS2 in these cell types [8, 51, 82, 108, 133, 146]. Furthermore, the up-regulation of immune system- and cell damage-associated genes accompany the SARS-CoV-2 infection of cultured neurons [125]. The latter is in agreement with the potential neurovirulence of SARS-CoV-2, as supported by studies of postmortem brain tissue [36, 52, 92, 117] and radiological brain studies [41]. Therefore, currently available data, although limited and still debated, suggest that neural infection with SARS-CoV-2 is plausible, at least under certain conditions, warranting further investigations aiming to establish whether SARS-CoV-2 can efficiently replicate within the CNS and, if so, how the replication and spread are affected by the intrinsic anti-viral responses of different cell types in the CNS.

Susceptibility of CNS cells to SARS-CoV-2 infection along with their ability to limit viral replication likely influence the probability and severity of neurological manifestations that vary between COVID-19 patients depending on age, comorbidities, and genetic predispositions.

One of the most vulnerable populations of COVID-19 patients is represented by individuals with Down syndrome. Among adult COVID-19 patients, the individuals with DS experience higher incidence of consciousness/confusion compared to general population, show a rapid increase of mortality rate after age 40, and die at higher rate than non-DS individuals [62, 104]. In addition, COVID-19 patients with DS younger than 18 have higher incidence of medical complications than patients of the same age group without DS [44]. The predisposition to Alzheimer’s disease (AD) (mainly due to triplication of the gene encoding for APP [40, 77]) and the chronic hyperactivity of the interferon system (primarily due to triplication of genes encoding for interferon receptor subunits IFNAR1, IFNAR2, and IFNGR2, IL10RB, and interferon-stimulated genes MX1and MX2 [61, 134]) may contribute to the increased rate of neurological symptoms in DS individuals who contracted the virus [62]. Although currently there is no direct link established between the T21-associated interferonopathy [76] and specific COVID-19 neurological symptoms in patients with DS, the constitutive interferon system activation had been proposed to underlie the post-acute sequelae of COVID-19 [141]. In addition, the triplication of Chromosome-21 (HSA21) genes that are directly or indirectly implicated in SARS-CoV-2 entry mechanisms (e.g., genes encoding for virus-priming protease TMPRSS2 [101] and for ACE2 transcriptional regulator DYRK1A [120]) may be associated with the differential outcomes of SARS-CoV-2 infection of euploid versus trisomic CNS cells.

We hypothesized that brain cells with trisomy-21 (T21) may be more susceptible to SARS-CoV-2 infection, compared to euploid (EUPL) cells, due to enhanced virus entry via the ACE2/TMPRSS2 mechanism. However, these cells may also have a stronger potential to dampen virus replication and spread due to the elevated interferon system activity. Furthermore, the interferon system hyperactivity in DS might have a complex multiphasic effect on the outcome of SARS-CoV-2 infection. Increased baseline expression and/or virus-induced up-regulation of several interferon-stimulated genes (ISGs) in neurons and glial cells in the CNS may limit direct infection at the acute phase of virus invasion. However, the chronic hyperactivity of the interferon system in CNS may exacerbate neuroinflammation in COVID-19 DS patients leading to more severe neurological outcomes.

To test this hypothesis, we established primary cortical cultures from human postmortem fetal cortical specimens [17, 18, 37, 38, 59, 63, 64, 73, 138] and compared the levels of infectivity between EUPL and T21 cultures by RT-qPCR amplification of viral Spike RNA. As we demonstrated earlier, after being maintained in vitro for prolonged periods of time, the cells isolated from cortical specimens exhibit properties of mature neurons, including presence of synaptic contacts between neurons [37], expression of KCC2 transporter [143], inhibitory GABAergic currents [73], and presence of all adult tau isoforms [38]. Moreover, long-term T21 cultures gradually develop Alzheimer disease-like pathological features [19, 20, 64, 103] and are useful as an in vitro model to investigate specific molecular mechanisms of AD in DS [17, 37, 59, 64, 145]. We confirmed the expression of genes involved in SARS-CoV-2 main entry mechanism (ACE2 and TMPRSS2) and interferon-stimulated genes (MX1, STAT1, and STAT2) in EUPL and T21 cultures as well as characterized the relationships between SARS-CoV-2 infectivity and expression levels of these genes. Furthermore, utilizing postmortem brain specimens from three groups of non-infected elder subjects (controls without AD, subjects with late-onset AD, and subjects with DS and AD (DS-AD)), we analyzed the expression of SARS-CoV-2 entry proteins (ACE2 and TMPRSS2) and proteins that reflect interferon system activity (STAT1 and STAT2) in the human entorhinal cortex region. Our findings suggest that multiple factors, including ploidy and chromosomal sex, may contribute to the SARS-CoV-2 infectivity in cortical cells at the earlier stages of infection.

## Materials and methods

### Primary cortical cultures generation and maintenance

Primary human cortical cultures were established from 12 EUPL (9 XX, 3 XY) and 6 T21 (2 XX, 4 XY) postmortem fetal specimens of the cerebral cortex procured at 15–20 weeks of gestation and according to previously published protocol [18, 19, 59, 63, 73, 138] with minor modifications as described below. The procedure for obtaining postmortem fetal cortical specimens complied with all federal and institutional guidelines. The demographic information of fetal tissue cases is summarized in Table 1 and the number of specimens and cultures used in the experiments are shown in Table 2. The cells were plated at high density (100,000 cells per cm^2^) into the PEI-covered culture plates in DMEM supplemented with 10% FBS media, which was (1) completely replaced with Neurobasal media supplemented with N2 4 h after plating and (2) gradually replaced with BrainPhys media supplemented with SM1 and Glutamax starting at day 7 after plating. The cells were maintained at 37^0^C and 5% CO_2_ and fed every 3–4 days, with the 50% of media refreshed at each feeding. Under these conditions, fully differentiated mixed cortical cultures composed of major cell types (neurons, astrocytes, microglia, and oligodendrocytes) were generated, and all experiments (treatments and infection) were performed using 28–30 DIV (days in vitro) cultures.

**Table 1:** Demographic information of fetal brain specimens with the corresponding gestation age and biological sex. Postmortem interval was < 24 h for all specimens

Group	N	Gestational age, weeks Mean ± s.e.m
**Euploid**	**12**	
XX XY	9	17.9 ± 0.5
	3	18.6 ± 0.7
**Trisomy-21**	**6**	
XX XY	2	19.5 ± 0.5
	4	17.5 ± 0.9

The number of Euploid and T21 specimens (XX and XY combined) used in the study are shown in bold

**Table 2:** The number of cortical specimens and individual cultures used in each experimental set

Experiment	Cultures by experimental group	Per experiment	
		Number of specimens used to generate cultures	Cultures (*N* per group)
**VSV-eGFP-SARS-CoV-2 Infection # 1** **Cell imaging and counting, **Fig. 1	**EUPL:** XX EUPL-1 XX EUPL-2 XY EUPL-3	3	3
	**T21:** XX T21-12 XX T21-13 XX T21-14	3	3
**VSV-eGFP-SARS-CoV-2 Infections #2 and #3** • **#2: eGFP—RT-qPCR and** • **#3: eGFP and Spike—RT-qPCR, **Fig. 2	**XX EUPL:** XX EUPL-2 XX EUPL-4 XX EUPL-1 (x2) XX EUPL-7 XX EUPL-8 XX EUPL-9 (x2) XX EUPL-10	7	9
	**XY EUPL:** XY EUPL-5 XY EUPL-6 (x3)	2	4
	**XX T21:** XX T21-12 (x3) XX T21-13 (x2)	2	5
	**XY T21:** XY T21-14 (x2) XY T21-15 XY T21-16 (x2)	3	5
**SARS-CoV-2** **Infection #4** **(2 time points)** • **Spike—RT-qPCR, **Figs. 3, 5	**XX EUPL:** XX EUPL-11 (x4)	1	4
	**XY EUPL:** XY EUPL-5 (x4) XY EUPL-6 (x2)	2	6
	**XX T21:** XX T21-12 (x5)	1	5
	**XY T21:** XY T21-14 (x4)	1	4
**Treatment with IFNs:** **(3 time points; 2—3 treatment groups), **Fig. 7	**XX EUPL:** XX EUPL-2 (x27) XX EUPL-17 (x9)	2	4
	**XY EUPL:** XY EUPL-3 (x27) XY EUPL-5 (x3)	2	3–4
	**XX T21:** XX T21-12 (x27)	1	3
	**XY T21:** XY T21-15 (x6) XY T21-18 (x2)	2	3–4
**Background gene expression in untreated cultures** **(ACE2** **TMPRSS2** **MX1** **STAT1 ** **STAT2)—RT-qPCR, Figs. 4, 6**	**XX EUPL:** XX EUPL-1 XX EUPL-2 (x2) XX EUPL-7 XX EUPL-8 XX EUPL-9 XX EUPL-10 XX EUPL-11 (x8)	7	14–15
	**XY EUPL:** XY EUPL-3 (x2) XY EUPL-5 (x9) XY EUPL-6 (x4)	3	10–15
	**XX T21:** XX T21-12 (x20) XX T21-13 (x2)	2	18–22
	**XY T21:** XY T21-14 (x15) XY T21-15 XY T21-16 XY T21-18 (x2)	4	16–19

### Treatments of cultures with interferons

The interferons (10 ng/mL IFNα, 1 ng/mL IFNβ, and 100 ng/mL IFNγ (STEMCELLS Technologies Inc., USA)) were reconstituted according to manufacture protocol, stored in small aliquots at – 80 °C, and added to culture media immediately after thawing at the final concentrations. The concentrations for treatments were within the ranges reported for in vitro studies. The control cultures were treated with equal volumes of vehicle solution (HBSS). 48 h after the first treatment, the cell pellets were collected from first group of cultures (“48 h” group), whereas other two groups underwent two (“6 days” group) and three consecutive treatments (“10 days”) prior to cell pellet collection. The pellets were fast frozen on dry ice and stored at – 80 °C.

### Infection of primary cortical cultures

The VSV-eGFP-SARS-CoV-2 (Vesicular stomatitis virus (VSV) encoding the SARS-CoV-2 Spike and expressing eGFP as a marker of infection—kindly provided by Dr. Sean P. J. Whelan, Washington University) has been previously described [25]. Cells were infected with VSV-eGFP-SARS-CoV-2 at MOI of 0.01 for 2 h at which point cells were washed and fresh medium provided. The cultures were then incubated for 24 h and 48 h at which point cells were either fixed with 4% PFA for eGFP immunofluorescence microscopic analysis, or lysed with TRIzol Reagent (Ambion, 15596018) for RT-qPCR analysis. Another set of human cortical cell cultures was infected with SARS-CoV-2 (isolate USA-WA1/2020, BEI. MOI = 0.01) and incubated at 37 °C, 5% CO_2_ for 2 h at which point cells were washed and fresh medium was provided. Cells were lysed with TRIzol Reagent at either 2 h or 48 h post-infection (p.i.) and RNA was collected for RT-qPCR analysis. Sham-infected cultures were used as controls.

### Cell counting

Prior to fixation of cultures infected with VSV-eGFP-SARS-CoV-2, eGFP-positive cells in live cultures were counted manually under a florescent microscope at 24 and 48 h p.i. by scanning through the entire well for each individual culture. Because primary cultures derived from different specimens have different cell density by the time of treatment/infection (30 DIV), the phase-contrast images of each culture were taken to calculate the total number of cells per well at the time of infection. The accuracy of live cell counting was confirmed by subsequent analysis of mounted PFA-fixed cultures using Zeiss AxioScan.Z1 slide canner (Carl Zeiss). The results are expressed as percentage of infected (eGFP +) cells in individual cultures.

### RT-qPCR

Total RNA was isolated using RNeasy Plus Mini Kit (Qiagen) for samples infected with VSV and Direct-zol RNA Miniprep (Zymo Research) for samples infected with SARS-CoV-2. cDNA was made using Applied Biosystems High-Capacity cDNA Reverse Transcription Kit (Thermo Fisher). qRT-PCR was performed in technical triplicates using Applied Biosystems SYBR™ Select Master Mix for CFX (Thermo Fisher) on CFX96 Touch System (Bio-Rad). The following cycling conditions were used: 50 ℃ for 2 min, 95 ℃ for 2 min, and then 45 cycles at 95 ℃ for 15 s, 52 ℃ for 15 s, and 72 ℃ for 30 s. All primer pairs were adapted from the earlier published studies and are listed in Table 3. The relative expression (RE) of viral eGFP and Spike genes, as well as other cellular genes in infected cultures, was calculated from Ct values followed by normalization to total RNA (RE = 2^−(Ct)^/RNA) per PCR reaction. Alternatively, the delta-Ct method, with the HPRT1 as housekeeping gene, was used for qPCR analysis of cellular genes (not shown). When data from several independent experiments were pooled, all values were normalized to a standard (common sample or mean of values from several common samples) that was ran in all of these experiments to adjust for a potential “batch effect.”

**Table 3:** Sequences of earlier published primers used for PCR amplification in this study: ACE2 [96], shACE2 [96], MX1 [96], TMPRSS2 [9], STAT1 [112], STAT2 [112], HPRT1 [96], GFP [4], SRY [32], FMR1 [55]

Primer name	Sequence
ACE2	Forward: 5′-GGGCGACTTCAGGATCCTTAT
	Reverse: 5′-GGATATGCCCCATCTCATGATG
shACE2	Forward: 5′-GGAAGCAGGCTGGGACAAA
	Reverse: 5′-AGCTGTCAGGAAGTCGTCCATT
MX1	Forward: 5′-ACCTGATGGCCTATCACCAG
	Reverse: 5′-TTCAGGAGCCAGCTGTAGGT
TMPRSS2	Forward: 5′-AGCTGCAGAAGCCTCTGACTTTC
	Reverse: 5′-AGCGTTCAGCACTTCTGAGGTC
STAT1	Forward: 5′-TGTATGCCATCCTCGAGAGC
	Reverse: 5′-AGACATCCTGCCACCTTGTG
STAT2	Forward: 5′-CCGGGACATTCAGCCCTTTT
	Reverse: 5′-CTCATGTTGCTGGCTCTCCA
HPRT1	Forward: 5′-TGACACTGGCAAAACAATGCA
	Reverse: 5′-GGTCCTTTTCACCAGCAAGCT
GFP	Forward: 5′-AAGCTGACCCTGAAGTTCATCTGC
	Reverse: 5′-CTTGTAGTTGCCGTCGTCCTTGAA
SRY	Forward: 5′-CATGAACGCATTCATCGTGTGGTC
	Reverse: 5′-CTGCGGGAAGCAAACTGCAATTCTT
FMR1	Forward: 5′-CCCTGATGAAGAACTTGTATCTC
	Reverse: 5′-GAAATTACACACATAGGTGGCACT

### Confirmation of biological sex of fetal specimens

DNA was isolated using TRIzol following manufacture protocol (Invitrogen™ 10296010). PCR was performed on Veriti 96-well thermal cycler (Applied Biosystems) to amplify SRY (Y chromosome) and FMR1 (X chromosome) transcripts. The following cycling conditions were used: 94 ℃ for 2 min; then 35 cycles at 94 ℃ for 15 s, 50 ℃ for 15 s, and 72 ℃ for 15 s; and then 72 ℃ for 10 min. The primers are listed in Table 3.

### Human brain specimens

The frozen human brain specimens were obtained from UCI-MIND Brain Tissue Repository in compliance with all federal and institutional regulations. Demographic characteristics of autopsy tissue are summarized in Table 4. No significant changes were found in the postmortem intervals between groups. The lysates were prepared from the entorhinal cortical region of each tissue sample and processed for analysis by Western blot or ELISA as described below.

**Table 4:** Demographic information of brain specimens with the corresponding postmortem intervals (PMI), ApoE status, and neurofibrillary stages [12]

Group	N	Age, years Mean ± s.e.m	PMI, hours Mean ± s.e.m	ApoE status	Tangle stage	Plaque stage
**Control**		**88.9 ± 1.6**	**4.3 ± 0.4**			
F M	5	89.8 ± 1.5	4.4 ± 0.5	2/3, 2/3, 2/3, 2/3, 3/3	I, II, II, III, IV	none
	5	88 ± 3.0	4.3 ± 0.5	2/3, 2/3, 3/3, 3/3, 3/3	I, II, II, II, II	none
**AD**		**89.8 ± 1.5**	**5.1 ± 0.5**			
F M	5	89 ± 1.2	4.1 ± 0.6	3/3, 3/3, 3/3, 3/4, 3/4	III, III, III, III, V	A, B, B, B, C
	5	90.6 ± 2.9	6.0 ± 0.7	3/3, 3/3, 3/4, 3/4, 3/4	II, III, III, IV, IV	A, B, B, B, C
**DS-AD**		**58.5 ± 2.6**	**4.3 ± 0.7**			
F M	5	57.4 ± 3.1	4.6 ± 0.8	3/3, 3/3, 3/3, NA, NA	VI, VI, VI, VI, VI	B, C, C, C, C
	5	59.6 ± 4.4	4.0 ± 0.5	3/3, 3/3, 3/3, 3/3, 3/3	VI, VI, VI, VI, VI	B, C, C, C, C

The Means and standard errors of the age and PMI for control, AD and DS-AD subjects (Males and Females combined) are shown in bold

### Western blot

Homogenates of brain tissue specimens were prepared in RIPA buffer with addition of Halt protein inhibitors cocktail (Thermo Scientific), 5 mM EDTA and 1 mM PMSF. Samples were boiled for 5 min in SDS loading buffer with 50 mM DTT and ran on 4–12% PAAG (GenScript). Each gel set was loaded with samples from all three experimental groups (no-AD controls, AD, DS-AD) including a common sample that was used as a standard. This standard sample was included in each gel and the values corresponding to each band in a gel were normalized to the value from the standard sample to prevent a potential “batch effect.” Each sample was run in duplicates; 6 independent gels were analyzed. After transfer onto PVDF membranes (Bio-Rad) and blocking in 5% fat-free milk/TBST for 1 h blots were incubated overnight at 4 °C in primary antibodies: anti-TMPRSS2 (EMD Millipore, MABF2158), 1:2000; anti-STAT1 and anti-STAT2 (Cell Signaling, 14994 T, 72604S), 1:2000; anti-ACTG1 (Bio-Rad, MCA5776GA), 1:40,000. Then membranes were incubated for 1 h at room temperature (RT) with secondary antibodies: goat-a-mouse-HRP, 1:10,000 (R&D systems) or goat-a-rabbit-HRP, 1:1500 (Cell Signaling). Membranes were incubated for 5 min in Supersignal West Pico solution (Thermo Fisher), and signals were detected by exposing to X-ray film (Mini Medical 90 film processor, AFP Imaging) or using ChemiDoc XRS system (Bio-Rad). Quantification of bands intensity was done using ImageJ (NIH) and the intensity of the band of interest was normalized to the corresponding intensity of the housekeeping ACTG1 protein signal.

### ELISA

ACE2 protein expression in brain tissue homogenates was measured using human ACE-2 ELISA Kit (Ray Biotech, cat# ELH-ACE2-CL) according to the manufacturer’s protocol. The signal was measured using an ELx808 Plate reader (BioTek). The ELISA plate was loaded with the samples from all three experimental groups (no-AD controls, AD, DS-AD). Two independently run ELISA plates were loaded and analyzed.

### Statistical analyses

All in vitro experiments were designed for side-by-side comparisons between experimental groups (cultures) maintained under standardized conditions. A total of 18 specimens (12 EUPL including 9 XX and 3 XY, and 6 T21 including 2 XX and 4 XY) were used to establish cell cultures. For in vitro studies, the number of specimens and individual cultures used in each experimental set are shown in Table 2. For brain tissue studies, an equal number of subjects were included in each group with demographics summarized in Table 4. For two-group comparisons, paired or unpaired T-test, or non-parametric Mann–Whitney U test were used; for multiple groups comparisons, one-way ANOVA with Tukey HSD test for post hoc analyses or non-parametric Kruskal–Wallis H test with post hoc Mann–Whitney U test were used. Shapiro–Wilk test was used to determine if normal distribution model fits the data. The outliers were identified by Tukey's fences method. For correlation analyses, the Pearson correlation coefficient (*r*) and *P* values were determined, and the results are reported in APA format. To investigate the interactions, Factorial ANOVA Unbalanced design was used. The *P* values were determined using validated in R online calculators http://www.statskingdom.com/. The means and standard errors were calculated using Excel software. One-way ANCOVA for 3 independent samples was used to adjust for concomitant variable.

## Results

### Primary human cortical cultures are susceptible to ACE2 receptor-mediated infection by VSV-eGFP-SARS-CoV-2 and SARS-CoV-2

**Fig. 1:**
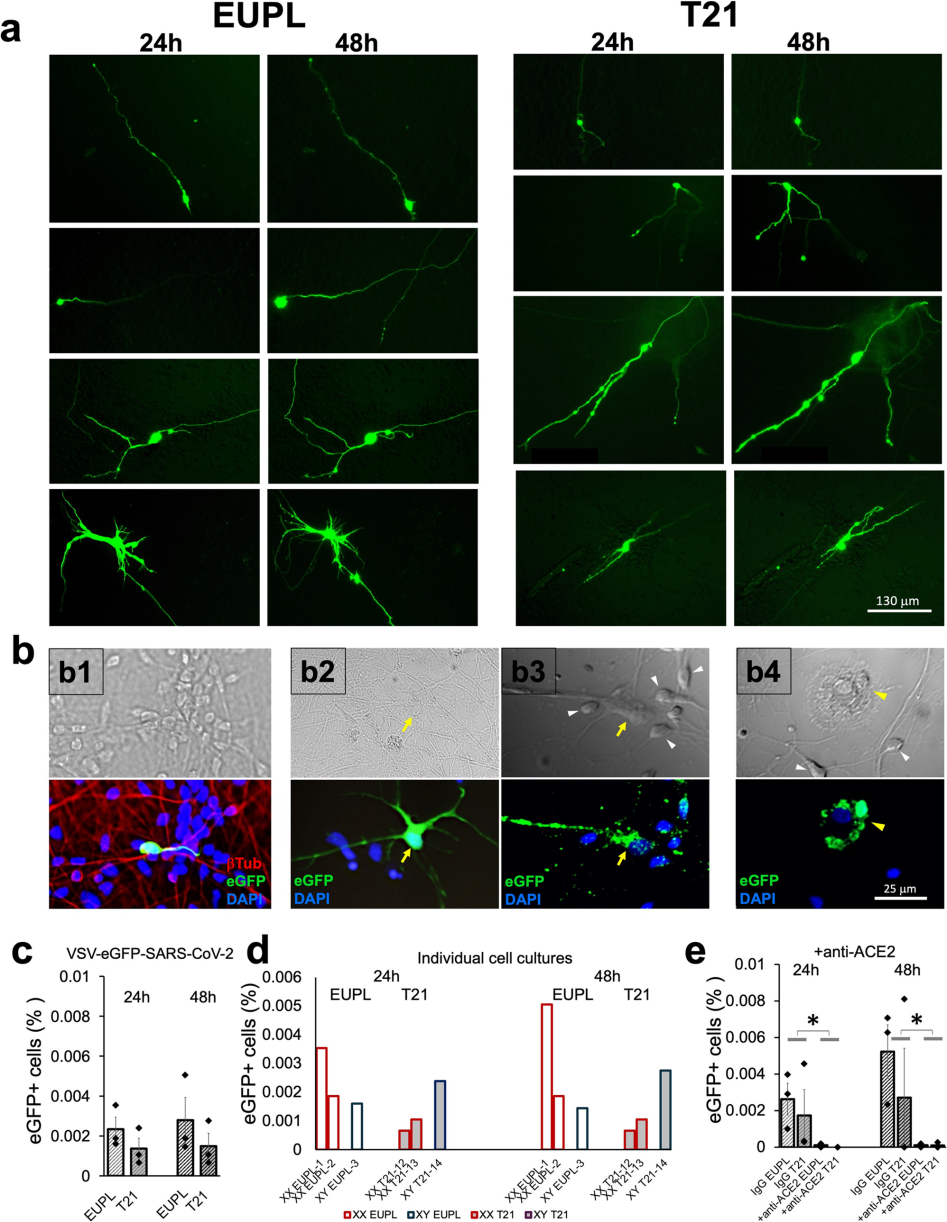
Spike-expressing VSV-eGFP-SARS-CoV-2 infects human cortical cells in vitro in an ACE2 receptor-dependent manner.** a** Representative eGFP-positive cells in mixed cortical cultures infected with VSV-eGFP-SARS-CoV-2 at 0.01 MOI and imaged at 24 and 48 h p.i. **b** Examples of eGFP fluorescence in fixed infected cells with morphology of neuron (b1), astrocytes (b2 and b3), and microglia (b4). Upper panels show transmitted light or DIC images; lower panels show fluorescence images of the corresponding regions. Co-localization of neuronal antibodies against β-Tubulin III and eGFP in infected neuron is shown in b1 (lower panel) and in Supplemental Fig. 1. Yellow arrows and arrowheads point out to cells exhibiting morphologic characteristics of cultured astrocytes and microglia, respectively. White arrowheads (b3, b4) point to cells exhibiting neuronal morphology. It should be noticed that the astrocytes in vitro acquire variable morphological features and are characterized by its larger, compared to neuronal, nucleus after labeling with DAPI (b3, lower panel). **c** Infectivity in euploid and T21 cultures by counting of eGFP-positive cells at 24 h and 48 h p.i. The absolute number of eGFP-positive cells per 421,500 cells (averaged total number of cells per well) was 15, 8, 7 (EUPL, 24 h); 3, 4, 10 (T21, 24 h); 21, 8, 6 (EUPL, 48 h); 3, 4, 12 (T21, 48 h). **d** Infectivity by cell counts in individual cultures derived from different specimens as indicated. **e:** Infectivity in control IgG-treated cultures versus in cultures treated with 20 mg/mL anti-ACE2 antibody (P(24 h) = 0.04002; P(48 h) = 0.04571; Pared T-test). Data shown as mean ± S.E.M

As a proof of concept, we first confirmed that primary cell cultures from human brain cortical specimens are susceptible to a SARS-CoV-2 infection. The cultures were infected with a Vesicular stomatitis virus (VSV) encoding the SARS-CoV-2 Spike protein and expressing eGFP as a marker of infection [25]. The presence of eGFP or Spike transcripts, as well as expression of ACE2 and the membrane protease TMPRSS2, was measured in cell pellets from infected cultures. In agreement with an earlier study that utilized human iPSC-derived neurons [95], infection with VSV-eGFP-SARS-CoV-2 revealed neurotropic properties in the primary cortical cultures, with many eGFP-positive cells exhibiting neuronal morphology (e.g., Fig. 1a, upper panels). As shown in Fig. 1a, the eGFP fluorescence was detected in cell bodies and processes already by 24 h p.i. and spread further along the processes during the next 24 h, similar to earlier findings in iPSC-derived neuronal cultures [95]. In addition to neuronal eGFP signals (Fig. 1b, panel b1 and Supplemental Fig. 1), the eGFP fluorescence was occasionally observed in cells morphologically reminiscent of astrocytes and microglia (Fig. 1b, panels b2, b3, and b4). As Fig. 1 (panels c, d, e) and Supplemental Table 1 show, the absolute number of eGFP-positive cells in each cell culture was low (with, on average, 3–15 cells per 421,500 cells in the individual culture). On average, T21 cultures had lower percentage of eGFP-positive cells compared to euploid cultures (both at 24 h and at 48 h p.i.), and the differences between the two groups were not significant possibly due to high level of variability between individual cultures (Fig. 1c, d). To establish whether infectivity of VSV-eGFP-SARS-CoV-2 in primary cortical cultures is mediated by ACE2 receptors, we treated a group of cultures with the anti-ACE2 antibodies and compared the infectivity in these cultures with the infectivity in the corresponding control IgG-treated sister-cultures. As shown in Fig. 1e, the anti-ACE2 antibody effectively blocked the VSV-eGFP-SARS-CoV-2 infectivity in both T21 and EUPL cultures, whereas IgG treatment alone produced no significant effect on the infectivity. These results support the ACE2 receptors-mediated mechanism of VSV-eGFP-SARS-CoV-2 infectivity.

**Fig. 2:**
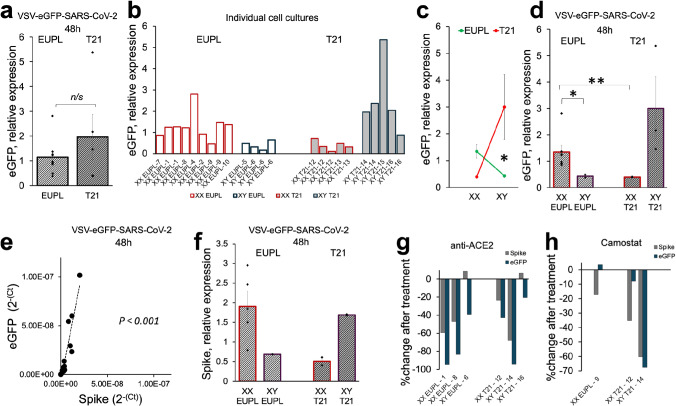
VSV-eGFP-SARS-CoV-2 infectivity by RT-qPCR in euploid and T21 cortical cultures is mediated by ACE2 receptors and may depend on chromosomal sex.** a** RT-qPCR amplification of eGFP transcript in EUPL and T21 cultures 48 h p.i. No significant differences between EUPL and T21 cultures are found when XX and XY groups are combined (*T*-test, *P* = 0.4311; *N*_EUPL_ = 9 specimens, *N*_T21_ = 5 specimens). **b** Infectivity in individual cultures derived from different specimens as indicated. **c** Significant interaction between ploidy and chromosomal sex of specimens contributes to the infectivity (factorial two-way ANOVA, *P* = 0.0311). **d** Comparisons between EUPL and T21 groups with stratification for chromosomal sex (*T*-test, *P < 0.05, **P < 0.01; *N*_XX EUPL_ = 7 specimens, *N*_XY EUPL_ = 2 specimens, *N*_XX T21_ = 2 specimens, N_XY T21_ = 3 specimens). **e** Significant large positive relationship between eGFP and Spike RNA transcripts amplified from the same samples in infected cultures (*r*(14) = 0.927, *P* < 0.001); shown are the cycle threshold (Ct) value data converted to a linear form. **f** RT-qPCR amplification of viral Spike RNA level produces results reminiscent of shown in panel “d.” **g** Percent reduction of infectivity measured by eGFP and Spike RNA at 48 h p.i. in the presence of 20 mg/mL anti-ACE2 antibody compared to infectivity in control IgG-treated cultures. **h:** Reduction of infectivity at 48 h p.i. in three cortical cultures after treatment with 50 μM of camostat mesylate; the percent reduction in eGFP and viral Spike RNA levels in camostat-treated cultures relative to that in corresponding vehicle-treated sister-cultures is indicated for each culture. For “**a, d, f**,” the data shown as mean ± S.E.M.; each data point is a value corresponding to individual cortical specimen from which the cultures were generated. The values from individual cultures (as shown in “b”) are averaged when more than one culture was generated from the same specimen

These findings were replicated in the next independent experiment, in which we used RT-qPCR to amplify the eGFP and Spike RNAs after infection of cultures with VSV-eGFP-SARS-CoV-2. Both transcripts were detected in infected cultures 48 h p.i. despite a low MOI. As shown in Fig. 2a, the expression of eGFP was not different between infected euploid and T21 cultures 48 h p.i. However, a high degree of variability was noticed between levels of infection in individual cultures (Fig. 2b), with the eGFP expression levels seemingly being associated with chromosomal sex of cells (e.g., XX versus XY). Based on PCR amplification of SRY and FMR1 gene loci on Y and X chromosomes, respectively (data not shown), the cultures were designated as “XX” or “XY.” Although our study was limited by a relatively small number of available cortical specimens from which the primary cultures were established, we performed an exploratory subgroup analysis in which the data points from individual cultures originated from the same specimen (sister-cultures) were averaged. This analysis revealed a significant interaction between ploidy and chromosomal sex in infected cultures (Fig. 2c) and showed significantly higher eGFP transcript levels in the XX EUPL group compared to the XX T21 group, as well as in the XX EUPL group compared to the XY EUPL group (Fig. 2d). Furthermore, the RT-qPCR amplification of viral Spike RNA revealed similar trends (Fig. 2e, f). Importantly, the amplification of the eGFP and Spike transcripts from the same samples showed significant correlation, albeit with the eGFP transcription rate being higher compared to the rate of Spike amplification (Fig. 2e) likely due to a closer proximity of the former to a VSV promoter [25, 131, 137]. In agreement with the results shown in Fig. 1e, the presence of anti-ACE2 antibodies in culture media before and during infection drastically inhibited infectivity (Fig. 2g) as evidenced by reduction in the eGFP expression (in all cultures) and Spike expression (in four out of six cultures) at 48 h p.i. Furthermore, susceptibility to infection was also reduced in cultures treated with the inhibitor of TMPRSS2, camostat mesylate as determined by eGFP and Spike transcript levels (Fig. 2h), although the degree of inhibition varied between cultures derived from different tissue specimens. Of note, camostat mesylate treatment also reduced the number of eGFP-positive cells (by count) only in two out of six VSV-eGFP-SARS-CoV-2-infected cultures at 24 h p.i. (data not shown). Thus, the presence of alternative mechanisms that employ virus priming by camostat-resistant protease(s) cannot be excluded.

Collectively, these data confirm that Spike-expressing VSV is capable of infecting human cortical cells in vitro via a mechanism that employs the ACE2 receptor and TMPRSS2 protease.

**Fig. 3:**
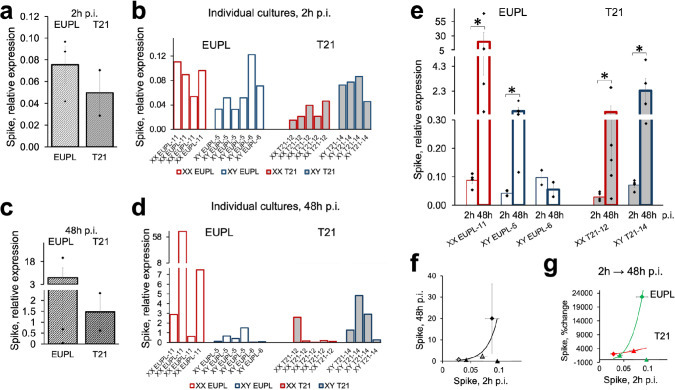
SARS-CoV-2 infectivity by RT-qPCR amplification of viral Spike RNA in EUPL and T21 cortical cultures 2 h and 48 h p.i.** a**, **c** Spike expression in cultures derived from EUPL and T21 specimens at 2 h p.i. (**a**) and 48 h p.i. (**c**) after infection with 0.01 MOI SARS-CoV-2. No significant differences between EUPL and T21 groups are found (T-test; *N*_EUPL_ = 3 specimens, *N*_T21_ = 2 specimens; each data point is a value corresponding to individual specimen. The values from individual cultures (as shown in “b” and “d”) are averaged when more than one culture from the same specimen was generated). **b, d** Infectivity in individual cultures derived from different specimens as indicated. **e** An increase in infectivity in four out of five sets of sister-cultures between 2 and 48 h p.i. (Mann–Whitney U test, *P < 0.05; data shown as mean ± S.E.M.; each data point is a value corresponding to individual culture derived from cortical specimen as indicated). **f, g** Infectivity measured by Spike RNA level at 48 h p.i. (**f**) and percent increase in Spike RNA level by 48 h p.i. (**g**) depend on the initial infectivity at 2 h p.i. in EUPL and T21 cultures. It should be noticed that one set of cultures out of five showed no increase in the Spike RNA level and was excluded from fitting

Although the entry mechanism of Spike-expressing VSV-eGFP-SARS-CoV-2 into cortical cells is presumably similar to that of SARS-CoV-2, the ensuing interferon response following infection is likely to be different compared to infection with authentic SARS-CoV-2 [7, 27, 111, 140]. We therefore infected cultures with SARS-CoV-2 (isolate USA-WA1/2020) at low MOI (0.01) and assessed the levels of viral infectivity by RT-qPCR amplification of Spike RNA transcripts at 2 h and 48 h p.i. Similar to VSV-eGFP-SARS-CoV-2, SARS-CoV-2 readily infected cortical cultures as evidenced by the RT-qPCR detection of Spike RNA in all infected cultures at 2 h p.i. On average, the level of Spike RNA at 2 h p.i. as well as at 48 h p.i. was lower in the T21 group compared to that in the EUPL group (Fig. 3a–d). Of note, consistent with the results of VSC-eGFP-SARS-CoV-2 infection, the SARS-COV-2 infectivity level at 48 h p.i. was, on average, higher in cultures derived from the XX EUPL specimen compared to those derived from XY EUPL specimens, whereas cultures derived from the XX T21 specimen had lower infectivity compared to cultures derived from the XY T21 specimen.

Importantly, between 2 and 48 h p.i., the level of Spike transcript increased in four out of five sets of sister-cultures (when comparing “2 h” vs. “48 h” sets of sister-cultures originated from the same specimen) (Fig. 3e). For these four sets of cultures, the higher the level of Spike RNA at the earlier time point (2 h), the higher the infectivity level at 48 h p.i., (Fig. 3f), with the percent increase in the level of infectivity during the period of time between 2 and 48 h p.i. being greater in EUPL cultures compared to that in T21 cultures (Fig. 3g). The remaining one set of cultures exhibited highest, among all sets, mean level of infection at 2 h p.i., with the subsequent reduction of Spike level by 43% at 48 h p.i. Given that cultures were exposed to the virus only for 2 h of inoculation (after which the virus was washed out), the increase in the Spike transcript level supports the likelihood of virus replication in the cells within the cultures, with the rate of replication being higher in EUPL than in T21 cultures.

Collectively, the results from experiments using VSV-eGFP-SARS-CoV-2 and patient SARS-CoV-2 isolates demonstrated that cortical cultures are susceptible to infection with these viruses and pointed out at potential interaction between ploidy and chromosomal sex that may contribute to infectivity levels in cortical cells.

### Differential expression of ACE2 and TMPRSS2 genes in non-infected and SARS-CoV-2 infected EUPL and T21 human cortical cultures

**Fig. 4:**
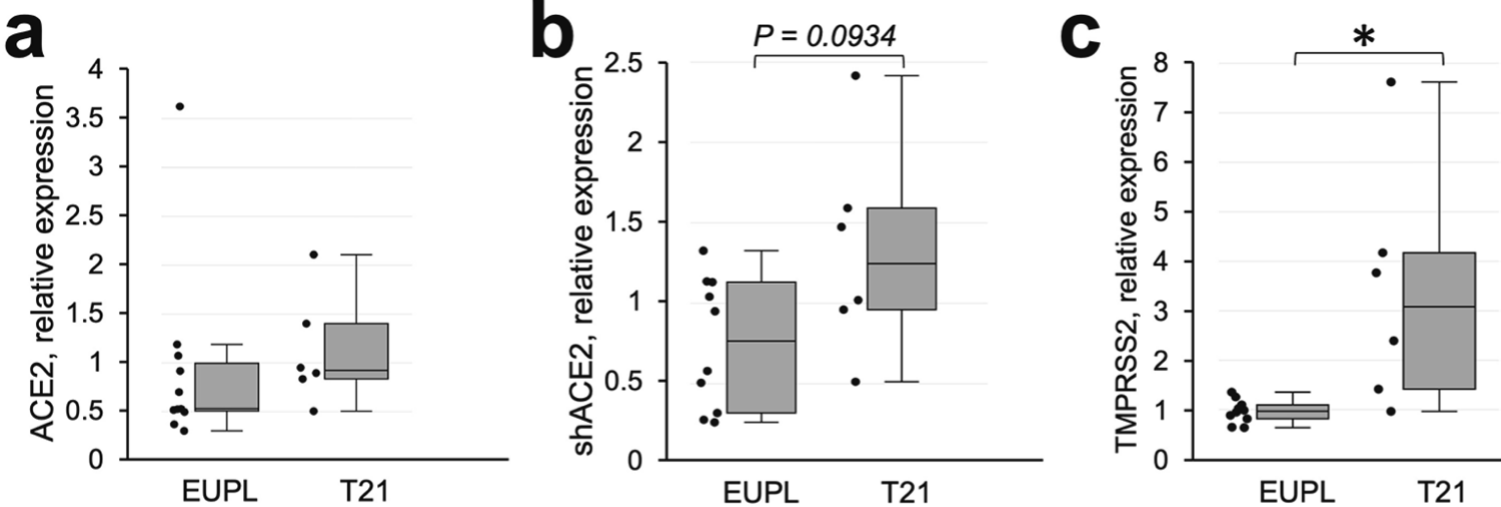
mRNA expression of ACE2 and TMPRSS2 in non-infected primary cortical cultures derived from EUPL and T21 specimens. **a**, **b** Relative mRNA expression of full-length ACE2 isoform (**a**) and short isoform shACE2 (**b**) in EUPL and T21 cultures by RT-qPCR. Two-group comparisons revealed no significant differences between EUPL and T21 specimens (Mann–Whitney U test, *P*_(ACE2)_ = 0.2561; *P*_(shACE2)_ = 0.0934). **c** TMPRSS2 expression by RT-qPCR is higher in T21 group compared to EUPL group (Mann–Whitney U test, *P_(TMPRSS2)_ = 0.0047). Each data point corresponds to an individual cortical specimen and is an average of values obtained from sister-cultures generated from this specimen. The data shown as first quartile, median (horizontal line within the box), third quartile, min and max

To gain insight into the origin of the differences in the infectivity levels between EUPL and T21 groups, we first compared their baseline expression levels of genes involved in SARS-CoV-2 main entry mechanism, specifically ACE2 and TMPRSS2. We then investigated how the experimental groups differ in their ability to transcriptionally regulate the expression of the indicated genes in response to SARS-CoV-2 infection. We also analyzed the relationships between the expression levels of these genes and the infection levels. RT-qPCR amplification reveals both a full-length, receptor-encoding ACE2 isoform, as well as a short ACE2 isoform (shACE2) in EUPL and T21 cortical cultures (Fig. 4a, b). Although the levels of ACE2 isoform expression were, on average, higher in the T21 group, the differences between the two groups were not significant. In contrast, the TMPRSS2 mRNA expression was higher in the T21 group compared to the EUPL group, which is in agreement with the gene dosage effect due to localization of the TMPRSS2 gene on HSA21 (Fig. 4c).

**Fig. 5:**
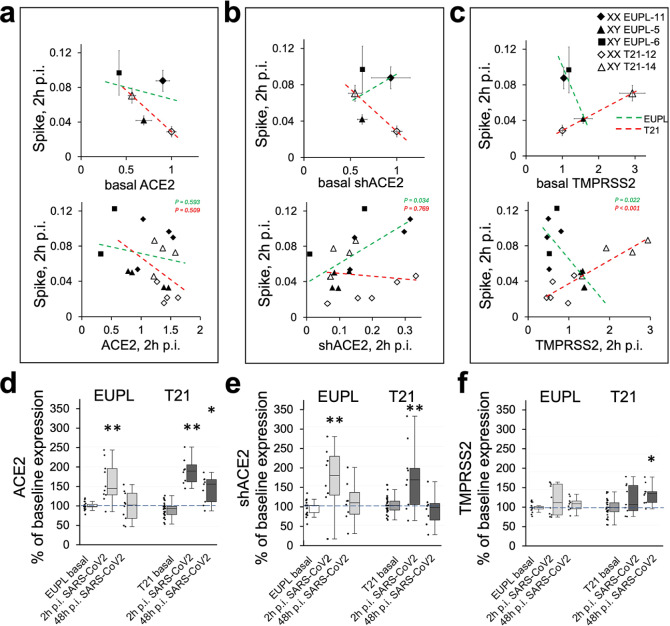
Relationships between SARS-CoV-2 infection level and mRNA expression levels of ACE2 and TMPRSS2 in cortical cultures.** a**–**c** Trends in the relationships between SARS-CoV-2 infectivity at 2 h p.i. and the basal (upper panels) or induced (lower panels) expression of ACE2 (**a**), shACE2 (**b**), and TMPRSS2 (**c**) in EUPL and T21 cultures generated from three EUPL and two T21 cortical specimens as indicated. The induced gene expression level and the Spike transcript level for each infected culture were measured in the same lysate from this culture, whereas the basal expression levels were measured in lysates from non-infected cultures and the values were plotted against Spike transcript values in the corresponding infected sister-cultures. **d**–**f** ACE2, shACE2, and TMPRSS2 mRNA expression levels in infected cultures relative to the mean baseline expression in the corresponding non-infected sister-cultures. The data shown as first quartile, median (horizontal line within the box), third quartile, min and max. The asterisks indicate that the differences between baseline and induced expression are significant (Mann–Whitney U test, *P < 0.05; **P < 0.01, statistical outliers identified by Tukey’s fences method are excluded when performing group comparisons)

To determine if differences in SARS-CoV-2 infectivity between groups are related to differential expression of ACE2 and/or TMPRSS2, we analyzed the trends in the relationships between the mRNA expression of these main entry factors and the expression of viral Spike RNA using cell lysates from the same or corresponding cortical cultures. As shown in Fig. 5a**,** the higher level of basal and induced ACE2 mRNA was associated with the lower level of infectivity in both EUPL and T21 groups. Given that cultures derived from specimen “XX T21-12” had the highest ACE2 expression level and exhibited lowest infectivity level among other cultures, the higher baseline ACE2 gene expression itself does not translate into a higher ACE2 receptor-mediated SARS-CoV-2 infectivity. The seeming contradiction in these data likely reflects the multifunctional nature of the ACE2 gene, which produces not only a receptor for Spike-expressing viruses but also belongs to a family of ISGs albeit the full-length receptor-producing ACE2 isoform had been characterized as a less responsive to the interferons and viruses compared to shACE2 that does not function as a receptor [10, 90]. Accordingly, the higher shACE2 basal expression in T21 cultures was associated with the lower infectivity (Fig. 5b, upper panel). However, a positive and significant relationship between shACE2 expression at 2 h p.i. and the infectivity in EUPL cultures was revealed (Fig. 5b, lower panel), suggesting virus-triggered induction of shACE2 with stronger effect in cultures that have lower basal shACE2 level (not shown).

The higher level of TMPRSS2 (Fig. 5c) was associated with the higher level of infectivity in T21 cultures. In EUPL cultures, in contrary, the higher level of TMPRSS2 was associated with lower infectivity, whereas lower TMPRSS2 expression was associated with high infectivity level.

To investigate whether ACE2 and/or TMPRSS2 expression levels in cortical cultures are affected by SARS-CoV-2 infection, we compared their mRNA expression in non-infected cultures with those in the corresponding infected cultures at two time points after exposure, 2 and 48 h p.i. Figure 5d–f summarizes the results of this analysis and demonstrates that both ACE2 isoforms are significantly, although transiently, up-regulated in SARS-CoV-2-infected EUPL as well as T21 cultures, whereas TMPRSS2 expression remains majorly unchanged in EUPL cultures and shows small but significant increase in T21 cultures 48 h p.i.

### Differential expression of anti-viral response genes in T21 and EUPL cortical cultures

**Fig. 6:**
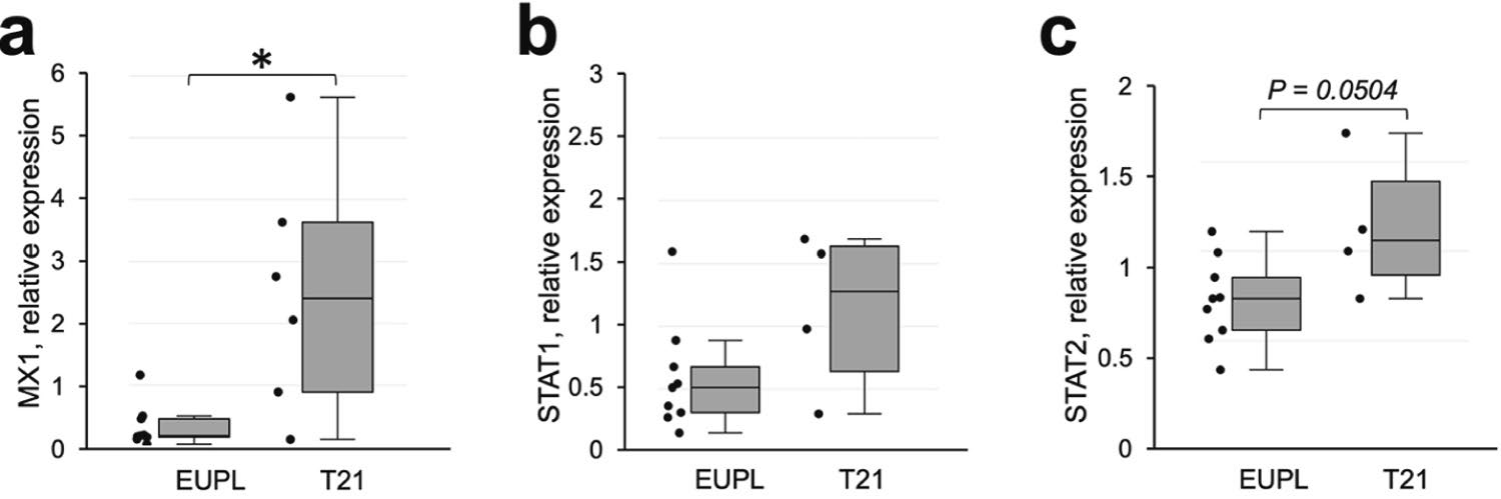
Expression of genes involved in interferon system activation pathway in non-infected primary cortical cultures derived from EUPL and T21 specimens. Relative mRNA expression of MX1 (**a**), STAT1 (**b**), and STAT2 (**c**) in T21 compared to EUPL group (Mann–Whitney U test, *P*_(MX1)_ = 0.0359; *P*_(STAT1)_ = 0.1986; *P*_(STAT2)_ = 0.0504). Each data point corresponds to an individual cortical specimen and is an average of values obtained from sister-cultures generated from this specimen. The data shown as first quartile, median (horizontal line within the box), third quartile, min and max

Given that ACE2 expression is sensitive to interferons [10, 90] and both isoforms of ACE2 gene were significantly up-regulated in SARS-CoV-2-infected cultures in our experiments, we reasoned that the expression level of ACE2 transcripts may be impacted by interferon responses in cortical cells. This hypothesis is supported by our observation that elevated ACE2 and shACE2 mRNA expression levels in non-infected XX T21 cortical cultures were associated with lower infectivity in the corresponding infected cultures, suggesting that elevated interferon system activity in XX T21 cultures at the time of introducing the virus might protect cells at the early stages of infection. We therefore compared the expression of the MX1, an interferon-sensitive gene located on HSA21, and the Signal transducer and activator of transcription-1 and 2 (STAT1 and STAT2) in our groups. In addition to being sensitive to IFNs [3, 144], STAT1 and STAT2 are key molecular components of the interferon receptors-mediated pathways that regulate transcription of ISGs. Because MX1 is triplicated in cells with T21, we tested whether its background expression in T21 cortical cultures differs from that in EUPL groups. As shown in Fig. 6a, T21 cortical cultures had significantly higher MX1 expression compared to EUPL cultures. The differences between EUPL and T21 groups in their mRNA expression of STAT1 and STAT2 were not significant, although both genes, on average, showed tendency to have higher expression level in cultures derived from T21 specimens compared with those derived from EUPL cortical specimens (Fig. 6b, c), consistent with elevated constitutive activity of the interferon system in the T21 group. These data support the notion that baseline interferon system activity in T21 cultures may play a gatekeeper function, limiting the infectivity of SARS-CoV-2 at the initial stages of infection.

### Primary human cortical cultures are sensitive to type-1 and type-2 interferons

We next reasoned that, in addition to differences in the mRNA levels for SARS-CoV-2 entry proteins and in the baseline interferon system activity between T21 and EUPL cultures, these cultures may also differ in their sensitivity to type-1 interferons and thus have unequal transcriptional responses to SARS-CoV-2. We therefore compared the sensitivity of T21 and EUPL groups to type-1 and type-2 interferons by measuring the induction of MX1 expression in cultures exposed to exogenous IFN-α/β and -γ. Although, the direct transcriptional effect of type-1 interferons on MX1 and STAT1/2 expression might be expected to peak between 2 and 16 h after the treatment [123], we tested if the ISGs expression levels will still be altered by 48 h after treatment, the time point matching the post-infection time point in the experiments described earlier. We also tested the effect of multiple treatments of cultures with interferons for a prolonged time (2 treatments/6 days; 3 treatments/10 days) to model the chronic interferon system stimulation in EUPL and T21 cultures.

**Fig. 7:**
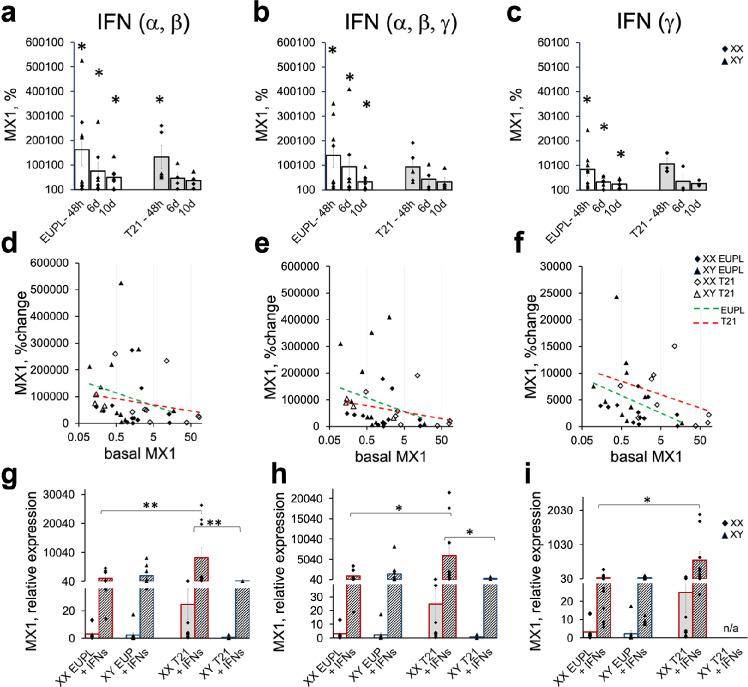
Interferons-induced up-regulation in the expression levels of MX1 in EUPL and T21 cortical cultures. **a**–**c** MX1 mRNA expression is up-regulated in cultures treated with **a** type-1 interferons (10 ng/mL IFNα and 1 ng/mL IFNβ), **b** a mixture of type-1 and type-2 interferons (10 ng/mL IFNα, 1 ng/mL IFNβ, 100 ng/mL IFNγ), and **c** with 100 ng/mL IFNγ alone for 48 h, 6d and 10d. Each data point is an expression level of MX1 mRNA in IFN-treated cultures shown in percent relative to vehicle-treated corresponding sister-cultures controls. Significant induction (%change) is indicated by asterisks (paired T-test comparisons of vehicle-treated vs. IFN-treated sister-cultures, *P < 0.05); **d**–**f** Non-significant small-to-medium negative relationships between baseline MX1 expression in vehicle-treated EUPL and T21 cultures and MX1 induction in the corresponding sister-cultures after treatment with IFNs (all time points (48 h, 6d, and 10d) are included)); **g**–**i** MX1 mRNA expression in cultures treated with IFNs α/β (**g**), IFNs α/β/γ (**h**), IFNγ (**i**), and in vehicle-treated corresponding sister-cultures. Data shown as mean ± S.E.M., with significant differences between IFN-treated groups indicated by asterisks (Kruskal–Wallis H test with post hoc Mann–Whitney U test, ^*^P < 0.05; **P < 0.01)

The exposure of cultures to a mixture of type-1 interferons (IFNαγ and IFNβ) for 48 h led to a significant up-regulation of MX1 gene in both EUPL and T21 cultures, with the effect being persistent, although typically attenuating after prolonged treatment for up to 6 days or 10 days (Fig. 7a). The concentrations of IFNs used for treating cultures were within the range of concentrations typically used for in vitro studies. In addition to type-1 interferons that are among the first cytokines produced in response to viral infection, the type-2 interferons are also involved in the anti-viral responses [80, 116]. Therefore, we added IFNγ to the culture medium 1.5 h after the type-1 interferons were added to investigate how the combination of type-1 and type-2 interferons affects the MX1 expression. We also tested whether IFNγ alone induces the expression of MX1 in EUPL and T21 cortical cultures. Similar to type-1 interferons, the combination of IFNα, IFNβ, and IFNγ produced up-regulation of MX1 in both EUPL and T21 cultures, although the increase in the expression at the earliest assessed time point (48 h) was somewhat dampened compared to the effect of type-1 IFNs, and only in EUPL cultures this effect was significant (Fig. 7b, c). Likewise, treatment of cultures with IFNγ alone produced induction of MX1, albeit much smaller than the type-1 IFNs induction, with the differences between basal and induced expression levels being significant only in EUPL group.

Collectively, these results confirm the responsiveness of cortical cells to type-1 and type-2 interferons and suggest that EUPL cultures might generate and sustain a stronger induction of ISGs in response to the virus-triggered interferon system as compared with T21 cultures. Of note, the degree of MX1 induction by IFNs (determined as the percent change relative to the corresponding baseline) tends to correlate negatively with the background MX1 expression (Fig. 7d–f), predicting that cultures with high background MX1 expression will exhibit weaker induction, thus inefficient clearance of virus that escapes anti-viral factors upon entrance, whereas cultures with lower background expression of MX1 will exhibit stronger induction of the interferon system, maintaining the anti-viral mechanisms that clear the infection. It is important to emphasize, however, that despite these apparent trends, the MX1 expression in XX T21 group after treatment with interferons was still significantly higher compared to that in XX EUPL and XY T21 treated cultures (Fig. 7g–i).

### SARS-CoV-2 infection induces up-regulation of ISGs in primary human cortical cultures

**Fig. 8:**
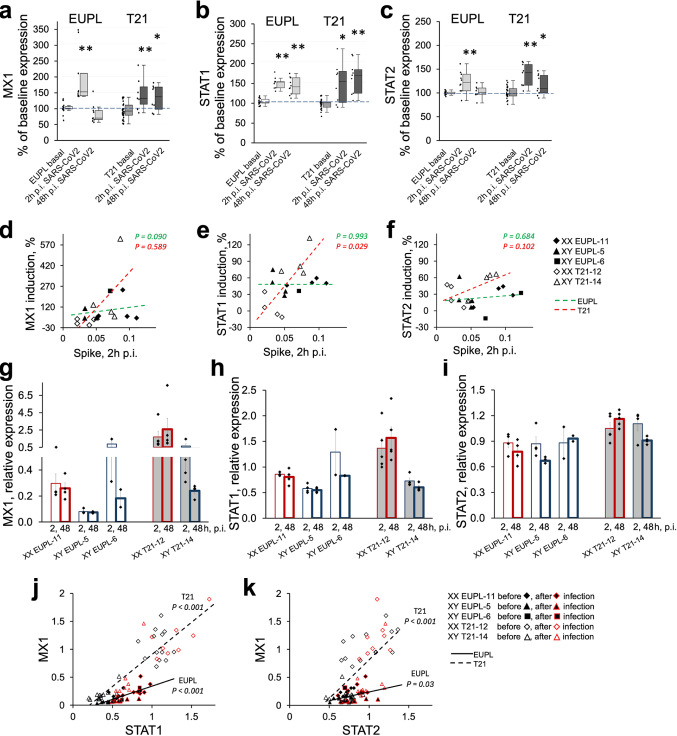
Induction of ISGs in EUPL and T21 cortical cultures infected with SARS-CoV-2.** a**–**c** mRNA expression levels of MX1 (**a**), STAT1 (**b**), and STAT2 (**c**) in infected cultures relative to the mean baseline expression in the corresponding non-infected sister-cultures. The data shown as first quartile, median (horizontal line within the box), third quartile, min and max. The asterisks indicate that the differences between baseline and induced expression are significant (Mann–Whitney U test, *P < 0.05; **P < 0.01, statistical outliers identified by Tukey’s fences method are excluded when performing group comparisons). **d**–**f** Relationships between SARS-CoV-2 infectivity (by RT-qPCR amplification of Spike transcript at 2 h p.i.) and induction (measured as percent change relative to baseline) of MX1 (**d**), STAT1 (**e**), and STAT2 (**f**) in EUPL and T21 cortical cultures. **g**–**i** Relative expression of MX1 (**g**), STAT1 (**h**), and STAT2 (**i**) in EUPL and T21 cultures 2 and 48 h p.i. Data shown as mean ± S.E.M.; each data point is a value corresponding to individual culture derived from cortical specimen as indicated. **j, k** Significant positive correlations between STAT1 and MX1 (**j**) and between STAT2 and MX1 (**k**) mRNA expression levels in individual EUPL and T21 cultures, both non-infected and SARS-CoV2-infected. Statistical outliers identified by Tukey’s method are excluded from fitting

To confirm that SARS-CoV-2 infection triggers an interferon system response in cortical cultures, we measured the induction of MX1 in SARS-CoV-2-infected cultures derived from the same cortical specimens as in Fig. 3, by comparing the MX1 mRNA expression levels between infected and corresponding non-infected sister-cultures. We also measured the SARS-CoV-2-induced changes in the expression levels of STAT1 and STAT2 mRNAs in these cultures. Of note, the STAT1 is an essential mediator of the virus-triggered cellular responses to interferons, including the early induction of MX1 [126], and itself contains an interferon-binding site at the promoter region [3, 144]. STAT2 interacts with STAT1 and is a critical component of the molecular pathway linking the virus-triggered type-1 interferon production with the induction of the ISGs. Figure 8a demonstrates that SARS-CoV-2 infection induces significant up-regulation of MX1 in EUPL and T21 cultures at 2 h p.i. Although by 48 h p.i., the expression of MX1 reduced in both EUPL and T21 groups, it maintained at significantly higher levels compared to baseline in SARS-CoV-2-treated T21 cultures but not in EUPL cultures. Interestingly, the STAT1 induction in SARS-CoV-2-infected cultures (Fig. 8b) was more persistent than that of MX1 in both EUPL and T21 cultures, with the significantly increased expression levels maintained by 48 h p.i. in both groups. In contrast, expression of STAT2 was similar to MX1, with a transient up-regulation in both groups at 2 h p.i., followed by partial recovery in EUPL cultures and was maintained up-regulated in T21 cultures by 48 h p.i. (Fig. 8c). The initial induction of MX1, STAT1, and STAT2 in T21 cultures infected with SARS-CoV-2 positively correlated with the level of viral Spike at 2 h p.i. (Fig. 8d–f). In contrast, there was only a very weak positive relationship between infection level, as measured by Spike expression, at 2 h p.i. and MX1 induction in SARS-CoV-2-infected EUPL cultures, and the induction of STAT1 and STAT2 in EUPL cultures at 2 h p.i. showed no dependence on the level of infection. Moreover, similar to the results seen after treatment of cultures with interferons, the amount of induction of ISGs (MX1, STAT1, and STAT2) at 2 h p.i. negatively correlated with the baseline level of their expression in corresponding non-infected sister-cultures (not shown), so that the cultures with higher background ISGs expression exhibited weaker ISGs induction. Further assessment of the MX1, STAT1, and STAT2 expression levels in SARS-CoV-2-infected cultures from EUPL and T21 cortical specimens consistently showed the highest, on average, expression of these genes in cultures derived from XX T21-12 specimen at both 2 h and 48 h p.i. time points (Fig. 8g–i).

Because the interferon-dependent transcriptional regulation of ISGs mechanistically relies on the availability of STATs, we next tested whether the MX1 expression was equally dependent on STAT1/STAT2 levels in EUPL and T21 cultures. As Fig. 8j, k demonstrates, the T21 cultures exhibit remarkably stronger correlations between STAT1 and MX1 expression (*R*^2^ = 0.7816 for T21 vs. *R*^2^ = 0.5421 for EUPL) and between STAT2 and MX1 expression (*R*^2^ = 0.392 for T21 vs. *R*^2^ = 0.1344 for EUPL) than do EUPL cultures, with the consistently higher slope of the linear regression fit in T21 compared to EUPL cultures, indicating higher rate of MX1 transcriptional regulation. Of note, the cultures cluster derived from XX T21-12 specimen, which had lowest infection level, has consistently the highest STAT1 and STAT2 expression, corresponding to the highest MX1 expression.

Altogether, our findings confirm that both EUPL and T21 cortical cells respond to SARS-CoV-2 with up-regulation of MX1, STAT1, and STAT2, consistent with virus-driven activation of interferon-mediated response in cortical cells. Furthermore, given that the expression levels of ISGs after infection in cultures derived from XX T21-12 specimen remained higher, on average, than in other cultures, it is compelling to hypothesize that persistently elevated activity of the interferon system (both constitutive and induced) in XX T21 cortical cultures may underlie a lower level of SARS-CoV-2 infectivity in this group. These findings may also imply that elevated interferon system activity in cortical cells of female DS COVID-19 patients may potentially provide stronger anti-viral response at early stages of infection but present a risk for neurological complications due to persistent immune hyperactivity at later stages.

### Differential expression of SARS-CoV-2 entry proteins (ACE2 and TMPRSS2) and STAT1/2 in entorhinal cortex of control, AD, and DS-AD individuals with no history of COVID-19

We next explored how our findings that utilized in vitro differentiated cortical cultures derived from fetal specimens could be applied to better understand and/or predict the potential clinical outcomes in COVID-19 patients with the risk for neurological complications. Specifically, we asked whether aged individuals with DS-AD have higher/lower levels of ACE2 and TMPRSS2 and/or STAT1 and STAT2 when compared to euploid individuals with or without AD. The protein homogenates were prepared from postmortem specimens of entorhinal cortex and subjected to Western blot analysis for detection of TMPRSS2, STAT1, and STAT2 or ELISA for ACE2 detection. Although the mean age in the DS-AD group (58.5 ± 2.6 years) was significantly younger, compared to age of control (88.9 ± 1.6 years) and AD (89.8 ± 1.5 years) groups (Table 4), we first performed analysis without adjustment for differences in age, given that premature aging is typical for individuals with Down syndrome [46, 102, 147] so that their “biological” age is likely to be more advanced than their “chronological” age. We then performed analysis of covariance to account for the age as a concomitant variable and repeated group comparisons after adjusting for age differences.

**Fig. 9:**
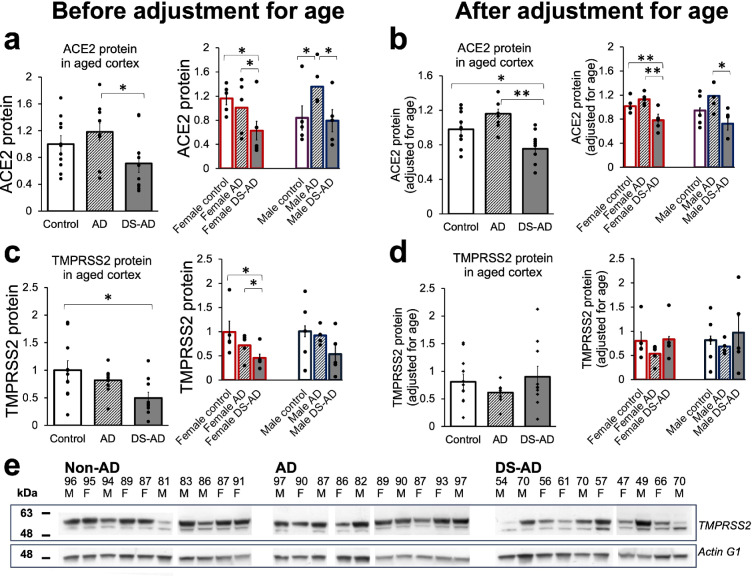
ACE2 and TMPRSS2 protein levels in entorhinal cortex of aged control, AD, and DS-AD subjects.** a, b** ACE2 down-regulation in DS-AD entorhinal cortex relative to AD and control groups before (**a**) and after (**b**) adjustment for differences in age (*Left* panels). *Right* panels: ACE2 protein levels in males and females of three groups. **c** TMPRSS2 down-regulation in DS-AD group compared to control and AD groups before adjustment for age (*Left* panel). *Right* panel: Significant reduction of TMPRSS2 in DS-AD female group compared to control females and AD female group. **d** After adjustment for age, the TMPRSS2, on average, is higher in DS-AD group compared to AD group, although no significant differences emerged for combined (*Right*) or stratified for sex (*Left*) group comparisons. Data shown as mean ± S.E.M., significant differences between groups are indicated by asterisks (ANOVA with post hoc Tukey HSD test for three-group comparisons; **P < 0.01; *P < 0.05). **e** Representative blots showing double band around 54 kDa consistent with the presence of partially processed full-length TMPRSS2 zymogen in the postmortem tissue

The protein level of ACE2 measured by ELISA in the DS-AD cortical specimens was significantly lower compared to that in AD group before and after adjusting for the differences in age. DS-AD group had also lower level of ACE2 than had the control group, although these differences were significant only after adjusting for age (Fig. 9a, b, *Left* panels). This reduced level of ACE2 in DS-AD relative to that in AD group was also significant when the data were analyzed separately for males and females. The reduction of ACE2 level in DS-AD relative to controls, however, was only revealed among females, but not among males (Fig. 9a, b, *Right* panels). In addition, the AD group had higher ACE2 level compared to control group, consistent with earlier reports [39, 146], although these differences were significant only between control males and AD males, whereas no differences were revealed between females in these two groups (Fig. 9a,* Right* panel).

The level of TMPRSS2 analyzed by Western blot (Fig. 9c–e) was also lower in DS-AD cortical specimens compared to that in AD and control groups, with the differences being significant between control and DS-AD groups (Fig. 9c). Further analysis with stratification for sex revealed significantly lower TMPRSS2 levels in DS-AD females compared to the corresponding control and AD groups (Fig. 9c,* Right* panel). After adjusting for age (Fig. 9d), the TMPRSS2 in DS-AD group was not statistically significant from other groups, although it tended to be higher compared to AD groups (Fig. 9d, *Left* and *Right* panels).

**Fig. 10:**
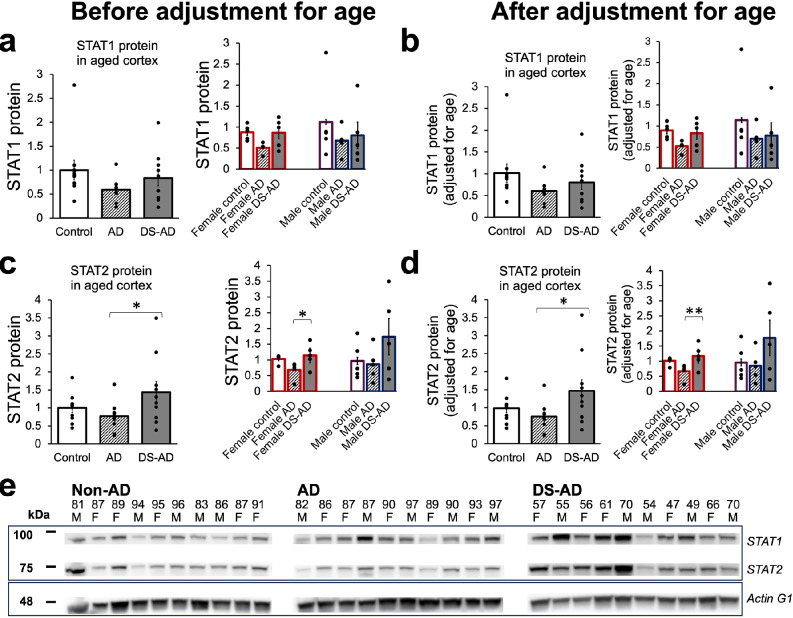
STAT1 and STAT2 protein levels by Western blot in entorhinal cortex of aged control, AD, and DS-AD subjects. Elevated STAT1 and STAT2 protein levels in DS-AD relative to the levels in AD groups before (**a**, **c**) and after (**b**, **d**) adjustment for age. Data shown as mean ± S.E.M., significant differences between groups indicated by asterisks (ANOVA with post hoc Tukey HSD test for three-group comparisons; **P < 0.01; *P < 0.05). **e** Representative Western blots of STAT1 and STAT2

Finally, we performed Western blot analysis of the STAT1 and STAT2 protein levels in the entorhinal cortex comparing control, AD, and DS-AD groups (Fig. 10a–e). When three groups were compared, the significant increase in the STAT2 level was detected in DS-AD group relative to AD group, although STAT1 level in DS-AD group was also, on average, higher than that in the AD group (Fig. 10a, c, *Left* panels). An increase in the STAT2 level was significant in females, but not in males DS-AD (Fig. 10c, *Right* panel). No other significant differences were revealed by three-group comparisons, and the adjustment for age did not change the outcomes of the results.

Collectively, comparisons of ACE2, TMPRSS2, STAT1, and STAT2 protein levels in postmortem specimens of entorhinal cortex from three groups of individuals suggest that ACE2 and TMPRSS2 protein levels are likely to be reduced in the DS-AD group compared to the AD or control groups. In contrast, STAT1 and STAT2 proteins are likely to be elevated in the DS-AD group, in particular in females, compared to the AD group, but not to the control group. These trends are revealed before adjusting for age and are more appropriate for the real-life circumstance, in which individuals with DS currently have life expectancy that is about 20 years shorter than in the general population [2, 14]. The reverse of the TMPRSS2 in DS-AD from being lower than in the other groups before adjusting for age to becoming higher after adjusting for age likely reflects age dependence of TMPRSS2 level and its overexpression in DS due to trisomy.

## Discussion

The potential ability of SARS-CoV-2 to infect and replicate in resident cells of the central nervous system (CNS) has been suggested based on multiple brain autopsy studies reporting the presence of viral protein and/or RNA in brain parenchyma and in sensory olfactory neurons [36, 45, 52, 86, 87, 106, 115, 119, 124]. However, the probability for COVID-19 patients to develop neurological symptoms, as well as the type and severity of symptoms, varies dramatically among individuals, pointing at the complex and multifactorial processes that determine neuroinvasiveness and regulate neurotropism of SARS-CoV-2.

Individuals with DS represent one of the most vulnerable populations of COVID-19 patients, with the increased risk of neurological complications and mortality [29, 62, 104] likely being a consequence of multiple comorbidities associated with HSA21 triplication. Among those are AD neuropathology [48, 49, 136], interferon system hyperactivity [28, 50, 76, 105, 122, 134], and immune deregulation, all of which compromise the ability of resident cells of the CNS to mount an effective immune response upon viral infection. The systemic immune deregulation associated with hyperactive but hyporesponsive interferon system was proposed to underlie higher severity of the infectious diseases including viral pneumonia in DS compared to general population [83]. It is possible that compromised integrity of the blood–brain barrier [68, 85, 98] due to chronic neuroinflammation associated with hyperactivity of interferon system and accumulation of AD pathology in DS increases the neuroinvasiveness of SARS-CoV-2, facilitating its entrance into the CNS. Once in the CNS, the virus can enter neurons and glial cells via several mechanisms, of which the ACE2 receptor-mediated entrance of furin- and TMPRSS2-primed virus at the cell’s plasma membrane is considered the most common and fastest [60, 75, 109]. The expression of both, ACE2 and TMPRSS2, can be affected by trisomy in DS given the HSA21 localization of the TMPRSS2 gene and transcriptional regulation of ACE2 by the HSA21-localized gene DYRK1A. Furthermore, due to global transcriptional dysregulation in DS, a plethora of genes present on other chromosomes can be up- or down-regulated [34, 35, 130].

Our study demonstrated the susceptibility of primary human cortical cultures to SARS-CoV-2. Although both eGFP fluorescence and eGFP/Spike RT-qPCR signals were readily detected after infection of cortical cultures with VSV-SARS-CoV-2 and SARS-CoV-2, the infection rates were low, consistent with the low MOI used in our experiments. Given that *in vivo*, even in airway epithelial cells, SARS-CoV-2 infections occur most likely at a low MOI and require multiple cycles of replication for the virus to spread [121], infecting the cortical cultures at low MOI may more accurately recapitulate the CNS viral infection than will infections at high MOI.

We also confirmed the expression of both ACE2 receptors and the TMPRSS2 protease in primary cultures developed from fetal cortical specimens and in aged brain samples of entorhinal cortex. Our results are consistent with earlier reports of the ACE2 and TMPRSS2 presence in the human prenatal brain [128] and in the adult human brain [56, 58, 82, 146]. Importantly, using cortical cultures, we were able to directly investigate the relationship between the expression levels of ACE2 and TMPRSS2 and the levels of infectivity of SARS-CoV-2 in euploid vs. trisomic cultures. Interestingly, despite that the anti-ACE2 antibody inhibited the Spike-expressing pseudovirus VSV-eGFP-SARS-CoV-2 infectivity, confirming the ACE2-dependent mechanism of entry, the cultures with the highest expression level of ACE2 (XX T21 group) had lowest infectivity. Given that the ACE2 gene is regulated by interferons [10, 90], the negative trend in the relationships between ACE2 expression and the level of infectivity may reflect increased interferon system activity in XX T21 cultures that likely plays an anti-viral role, limiting the level of infection in this group. This possibility is further supported by the increased level of other ISGs (MX1, STAT1, and STAT2) in XX T21 group of cultures both non-infected and SARS-CoV-2 infected.

In addition to extensively described mechanism of SARS-CoV-2 infection via ACE2 receptors and TMPRSS2 protease at the host cell’s plasma membrane [60], several other mechanisms involving receptors neuropilin-1 [22] and membrane proteases TMPRSS11A and TMPRSS11D [6], as well as the lysosomal/endosomal protease cathepsin L [11, 99, 114], have been implicated in facilitating the viral cell entry [5]. Although our experiments confirmed the importance of ACE2 and TMPRSS2 for the infection of cortical cells with a Spike-expressing virus, we cannot exclude the existence of additional alternative mediators/mechanisms facilitating the infection of cultures with SARS-CoV-2, especially given that the degree of reduction of the infection, particularly by the TMPRSS2 inhibitor camostat, varied between cultures. The alternative mechanism of entry via endocytosis [15, 66, 132] might involve endosomal enzymes (e.g., cathepsin L) that had been shown to cleave coronaviruses [11, 15, 114] facilitating the fusion. Given that the SARS-CoV-2 entry mechanism is largely determined by the availability of the priming proteases (TMPRSS2 or cathepsins) on target cells [99], the differences in TMPRSS2 expression levels between groups of cultures (with the highest expression in XY T21) may suggest that those cells that rely heavily on TMPRSS2-mediated entry will be more sensitive to camostat inhibition, whereas those that are employing TMPRSS2-independent priming will be relatively resistant to it. Although the latter mechanism of SARS-CoV-2 entry may contribute to infection of cortical cells, its efficiency in T21 cells is likely to be diminished due to an altered activity of lysosomal enzymes caused by accumulation of the APP-βCTF[69] and/or overexpression of cystatin-B [139], an endogenous inhibitor of cathepsin-B and -L [26, 33, 127]. Our results are consistent with the scenario in which high TMPRSS2 expression supports more efficient SARS-CoV-2 entry and is associated with high level of infectivity sensitive to camostat inhibition [109], whereas cultures with lower TMPRSS2 levels may rely on less efficient alternative mechanism (which might be further dampened in T21 cells) that is less sensitive to camostat. Alternatively, an apparent incomplete block of infection by ACE2 antibody and TMPRSS2 inhibitor might be a consequence of cell-to-cell spread of virus that occurred after inhibitors were removed from the culture media. Indeed, our first experiment that employed cell counting showed an increase in the number of infected cells in cultures from XX EUPL-1 and XY T21-14 specimens, suggesting the possibility for a secondary infection. Accordingly, four out of five sets of cultures infected with SARS-CoV-2 showed increased Spike RNA expression at 48 h p.i. compared to its expression level at earlier time point (2 h p.i.).

Our exploratory analysis uncovered the interaction between cell’s chromosomal sex and ploidy, with higher infectivity in XX EUPL and XY T21, but lower infectivity in XY EUPL and XX T21 cultures. Although to fully understand the potential impact of this interaction on SARS-CoV-2 infectivity in cortical cells will require further investigation, the differences between groups of cultures in their expression levels of TMPRSS2 as well as in the expression of several ISGs offer some possible explanations. At least in the T21 cultures, the infectivity level might be affected (1) by the expression of TMPRSS2, with higher availability of this protease being associated with the higher infectivity level, and (2) by the interferon system activity, with higher expression of ISGs (MX1, STAT1, and STAT2) being associated with lower infectivity level. The EUPL cultures in our experiments expressed lower level of TMPRSS2, compared to T21 cultures, suggesting that TMPRSS2-independent endocytotic mechanism of virus entry [15, 66, 132] might be more prevalent in the former group. The differences in the SARS-CoV-2 infectivity in EUPL cultures (XX vs. XY) could be related to sex differences in the mechanism of virus endocytosis. Although we are not aware of any study that would specifically investigate such differences in neural cells, numerous differences between male and female cells in phagocytosis, autophagy, and endocytosis have been reported [74, 91, 94, 142] and some of these differences might be related to the virus entry mechanism. Furthermore, several molecules including TLR7, TLR8, IL2RG, NKRF, DDX3X, UTX, EPAG, FOXP3, and IKKG that can impact type-1 IFN responses are encoded by genes located on the X chromosome [107], warranting a closer look at the contribution of biological sex to the anti-viral response in neural cells upon SARS-CoV-2 infection. At present, any conclusions drawn from our results pointing to a possible contribution of biological sex in regulating infectivity and/or anti-viral response should be considered tentative given a relatively low number of specimens from which the primary cultures were generated.

Our results showed that human cortical cultures in vitro readily respond to the exogenous type-1 and type-2 interferons, as well as to SARS-CoV-2 by up-regulating interferon-stimulated genes. The effect of exogenous interferons on ISGs expression likely involves not only the direct effect of IFNs, which typically peaks within the first few hours after exposure [80, 123], but also a secondary effect triggered by IFN-induced proinflammatory cytokines. The induction of MX1 and STAT2 was transient in SARS-CoV-2-infected cultures, with a significant increase at 2 h p.i. followed by a reduction, whereas the STAT1 transcript remained elevated in both EUPL and T21 cultures at the 48 h p.i. time point. This observation is consistent with the type-1 interferon-dependent induction of MX1 in primary cortical cultures. The significant positive correlations between STATs and MX1 expression, with the highest STAT1 and STAT2 expression levels corresponding to the highest MX1 expression detected in cultures with lower infectivity (XX T21), further corroborate the importance of type-1 interferon signaling in anti-viral response of cortical cells. Moreover, it suggests that elevated interferon system activity may play a protective role, limiting the level of infectivity at the early stages after infection. The amount of MX1 induction in response to type-1 interferons tended to be weaker in those cultures that had higher basal MX1 expression, and, in the SARS-CoV-2-infected cultures, was dependent on the level of infectivity. Thus, cultures with lower basal activity of interferon system (or lower expression of ISGs) had higher infectivity at 2 h p.i. but exhibited stronger induction. Despite this, the ISGs expression levels were still higher in T21 cultures, with the highest levels in cultures derived from XX T21-12 specimen. If the same scenario recapitulates in COVID-19 patients, the female patients with DS might have a lower virus load at the initial stages but be at higher risk for developing prolonged hyperactivation of immune system with the potential exacerbation of neuroinflammation and subsequent neurological complications.

To get an insight on whether cerebral cortical cells in individuals who are potentially vulnerable to neurological complications of COVID-19 (e.g., DS persons) differ from less vulnerable group in their expression of main SARS-CoV-2 interactors and/or the ISGs, we compared the protein levels of ACE2, TMPRSS2, STAT1, and STAT2 in homogenates prepared from postmortem specimens of entorhinal cortex of non-AD aged controls, age-matched subjects with AD, and DS-AD subjects. Here, we have chosen to analyze the specimens from un-infected subjects as we specifically aimed to investigate the baseline expression of the relevant proteins. Future studies of the specimens from individuals deceased from COVID-19 should gather additional knowledge and collectively with our current results provide a broader understanding of the diverse and dynamic nature of host–virus interactions in different groups of patients. We have selected entorhinal cortical region as it receives direct projections from the olfactory tract, and therefore, it is localized within the neural circuit that is most accessible for neuroinvasive viruses. In fact, in a rhesus monkey model, SARS-CoV-2 has been shown to spread from nasal mucosa to olfactory bulbs to entorhinal cortex, before the viral RNA detection in thalamus, medulla, and hippocampus [70]. The same study demonstrated that ACE2 receptors were highly expressed in olfactory bulbs and entorhinal area, further supporting the possibility of SARS-CoV-2 invasion of entorhinal cortex via olfactory route [70]. We presented the results both prior and after adjusting for difference in age because our DS-AD specimens were obtained from subjects that died at significantly younger age than control and AD subjects. Given the premature aging and shorter life expectancy of individuals with DS [46, 102, 147], mainly due to the near full penetrance of AD dementia [48, 49, 65, 135], the results presented without adjusting for age should more accurately reflect their true status of protein expression relative to other groups. We showed that both ACE2 and TMPRSS2 levels were significantly reduced in cortical homogenates of DS-AD females compared to AD and control females. The ACE2 and TMPRSS2 levels in DS males were also lower compared to that in AD males, although the differences in TMPRSS2 levels were not significant. These results may suggest that the conventional SARS-CoV-2 entry mechanism is less prevalent in DS-AD than in AD subjects but may also reflect faster enzymatic degradation and shedding. We also cannot exclude the selected loss of ACE2/TMPRSS2 expressing cells in the entorhinal cortex of DS-AD subjects which would reduce the levels of this proteases in the homogenates. Additional retrospective studies, utilizing cortical specimens from individuals with a confirmed COVID-19 history, are required to establish whether ACE2 and/or TMPRSS2 protein expression levels are associated with the infectivity and neurological complications in different categories of aged individuals. Our results revealed slight up-regulation of ACE2 protein level in entorhinal cortical samples of AD subjects compared to controls without AD, which is generally in agreement with earlier publications showing increased ACE2 levels in various brain regions in AD subjects [39, 110, 146]. However, in addition to previous publications, we observed that ACE2 level in the entorhinal cortex was increased in male AD, but not in female AD subjects, a finding possibly related to differences in the data analysis (stratified for sex vs. sexes combined) or in the choice of brain region used in our vs. other studies [31, 39, 110, 146].

The tendency for up-regulation of STAT1 and STAT2 protein levels in the entorhinal cortex of the DS-AD group, with the significantly higher STAT2 in DS-AD females compared to AD and control females, is consistent with increased background activity of the interferon system and up-regulation of ISGs in DS, which, in turn, may limit the infectivity levels at the initial stages of infection. It remains to be elucidated whether or not the levels of constitutively phosphorylated STATs are also elevated in the DS-AD entorhinal cortex as we were unable to detect the p-STAT1 using the available antibodies (data not shown). It had been proposed that DS has a feature of both, hyperactivity of immune system and immunosuppression [83], with the potential for initial virus protection but an increased risk for complications. As our in vitro results showed, the induction of ISGs, which is an essential part of the anti-viral cellular response, is dampen in cultured cells that have high constitutive interferon system activity. Therefore, the induction of ISGs in DS-AD entorhinal cortex in response to SARS-CoV-2 will likely be reduced, potentially resulting in prolonged infection.

It is important to note that this study, heavily based on results from in vitro experiments, has several limitations and therefore needs to be expanded to analyze brain samples from COVID-19 patients, as well as to further confirm the in vitro findings utilizing a larger cohort of cortical specimens, a broader range of MOI over the time course of infection, and evaluating cell-specific responses to SARS-CoV-2 exposure. Furthermore, this study is focused on RT-qPCR analyses of genes, but not the corresponding proteins, critically involved in regulation of the SARS-CoV-2 infectivity in vitro. Although this approach provides a fast and quantitative tool that potentially can be used for disease prognosis, it should be complemented by future studies of protein expression levels of SARS-CoV-2 interactors.

In conclusion, the susceptibility of human cortical cells to SARS-CoV-2 is likely determined by a complex interaction of multiple factors, including availability of entry proteases and the background activity of the interferon system. In turn, these factors can have differential distribution depending on age, brain region, and cell’s chromosomal sex. The expression levels of TMPRSS2 and ISGs in the CNS may determine the level of infection and thus affect the vulnerability of individuals with DS to neurological complications.

## Supplementary Information

Below is the link to the electronic supplementary material.

Supplementary file1 (TIF 7967 KB)

Supplementary file2 (PDF 1277 KB)

Supplementary file3 (PDF 130 KB)

## Data Availability

Data supporting the findings described in this study are available from the corresponding author upon reasonable request and execution of an institutional data transfer agreement.
