# Supplementary figures and images for: SARS-CoV-2 infection of human cortical cells is influenced by the interaction between aneuploidy and biological sex: insights from a Down syndrome in vitro model
Source: Acta Neuropathol. 2025 May 30;149(1):54. doi: 10.1007/s00401-025-02895-2 (PMC12125050; doi:10.1007/s00401-025-02895-2)

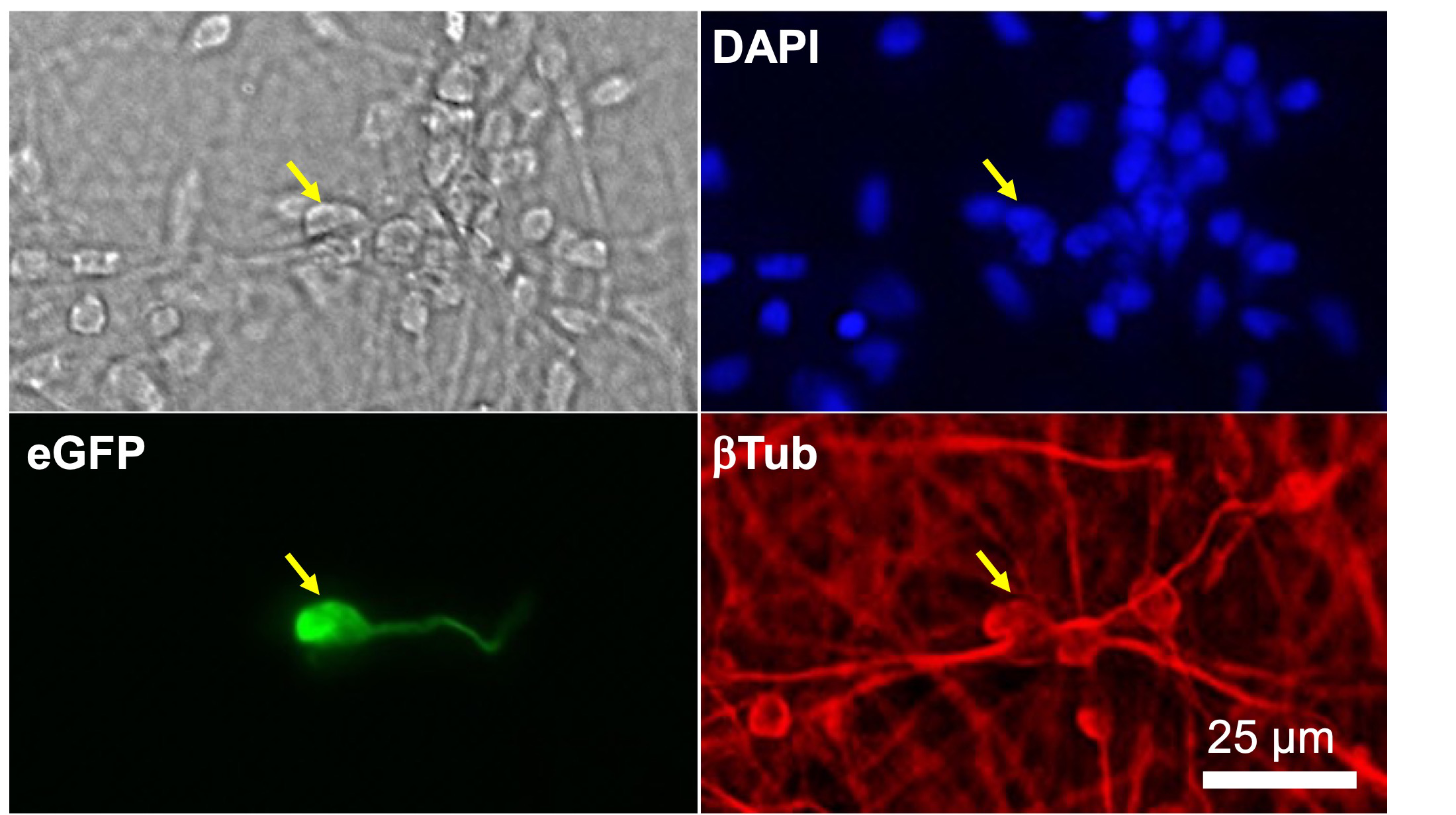

Supplement: Supplementary file 1 — Supplementary file1 (TIF 7967 KB) [file 401_2025_2895_MOESM1_ESM.tif]
