# Supplementary material for: SARS-CoV-2 infection of human cortical cells is influenced by the interaction between aneuploidy and biological sex: insights from a Down syndrome in vitro model
Source: Acta Neuropathol. 2025 May 30;149(1):54. doi: 10.1007/s00401-025-02895-2 (PMC12125050; doi:10.1007/s00401-025-02895-2)

Article title: SARS-CoV-2 infection of human cortical cells is influenced by the interaction between aneuploidy and biological sex: Insights from a Down syndrome *in vitro* model
Journal name: Acta Neuropathologica
Author names: Maria I. Lioudyno, Evgueni A. Sevrioukov, Gema M. Olivarria, Lauren Hitchcock, Dominic I. Javonillo, Sydney M. Campos, Isabel Rivera, Sierra T. Wright, Elizabeth Head, Juan Fortea, Thomas Wisniewski, A. Claudio Cuello, Sonia Do Carmo, Thomas E. Lane, Jorge Busciglio
Affiliation and e-mail address of the corresponding author:
Jorge Busciglio, Ph.D.
Department of Neurobiology & Behavior, UC Irvine
Email: jbuscigl@uci.edu

Supplemental Fig. 1 The transmitted light as well as individual fluorescence channels images of VSV-eGFP-SARS-CoV-2-infected cortical culture shown in Figure 1b (panel b1). The images were taken from PFA-fixed cultures labeled with anti-β-Tubulin III antibody to visualize neurons and DAPI stain for nuclei

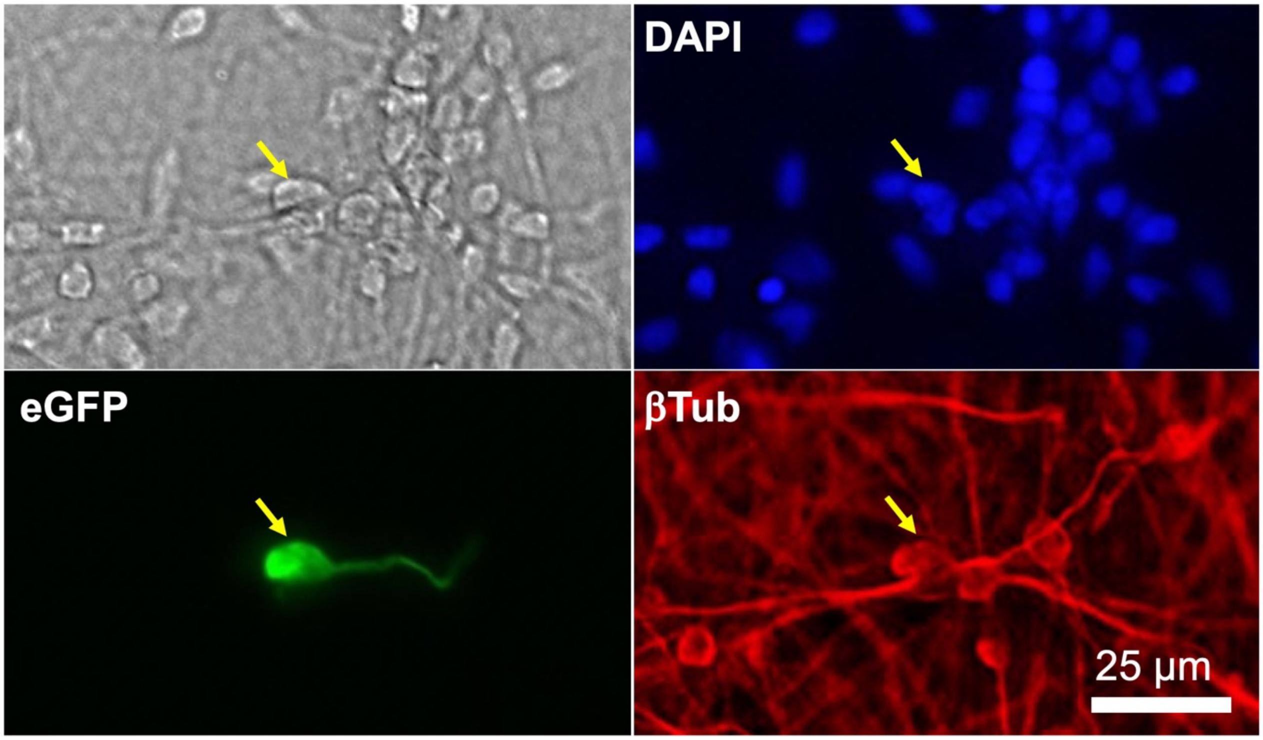

Supplement: Supplementary file 2 — Supplementary file2 (PDF 1277 KB) [file 401_2025_2895_MOESM2_ESM.pdf]
