# Supplementary material for: SARS-CoV-2 infection of human cortical cells is influenced by the interaction between aneuploidy and biological sex: insights from a Down syndrome in vitro model
Source: Acta Neuropathol. 2025 May 30;149(1):54. doi: 10.1007/s00401-025-02895-2 (PMC12125050; doi:10.1007/s00401-025-02895-2)
Supplement: Supplementary file 3 — Supplementary file3 (PDF 130 KB) [file 401_2025_2895_MOESM3_ESM.pdf]

Article title: SARS-CoV-2 infection of human cortical cells is influenced by the interaction between aneuploidy and biological sex: Insights from a Down syndrome *in vitro* model
Journal name: Acta Neuropathologica
Author names: Maria I. Lioudyno, Evgueni A. Sevrioukov, Gema M. Olivarria, Lauren Hitchcock, Dominic I. Javonillo, Sydney M. Campos, Isabel Rivera, Sierra T. Wright, Elizabeth Head, Juan Fortea, Thomas Wisniewski, A. Claudio Cuello, Sonia Do Carmo, Thomas E. Lane, Jorge Busciglio
Affiliation and e-mail address of the corresponding author:
Jorge Busciglio, Ph.D.
Department of Neurobiology & Behavior, UC Irvine
Email: jbuscigl@uci.edu

Supplemental Table 1. Number of eGFP-positive cells counted in cortical cultures 24 and 48h after infection with VSV-eGFP-SARS-CoV-2 at 0.01 MOI

Culture ID		Treatment group	Total number of cells per well at the time of infection	Number of eGFP+ cells per well		% of eGFP+ cells per well	
				24h p.i.	48h p.i.	24h p.i.	48h p.i.
**EUPL**	XX EUPL-1	Virus alone	594000	**21**	**30**	0.003535	0.005050
		+ IgG	722000	21	51	0.002909	0.007064
		+anti-ACE2	612000	0	0	0	0
	XX EUPL-2	Virus alone	428000	**8**	**8**	0.001869	0.001869
		+ IgG	601000	6	14	0.000998	0.002329
		+anti-ACE2	533000	1	1	0.000188	0.000188
	XY EUPL-3	Virus alone	622000	**10**	**9**	0.001608	0.001447
		+ IgG	781000	31	49	0.003969	0.006274
		+anti-ACE2	974000	1	1	0.000102	0.000102
**T21**	XX T21-12	Virus alone	151000	**1**	**1**	0.000662	0.000662
		+ IgG	299000	1	0	0.000334	0
		+anti-ACE2	270000	0	0	0	0
	XX T21-13	Virus alone	190000	**2**	**2**	0.001053	0.001053
		+ IgG	347000	1	0	0.000288	0
		+anti-ACE2	204000	0	0	0	0
	XY T21-14	Virus alone	544000	**13**	**15**	0.002390	0.002757
		+ IgG	592000	27	48	0.004561	0.008108
		+anti-ACE2	768000	0	2	0	0.000260
